# Photobleaching and Recovery Kinetics of a Palette of Carbon Nanodots Probed by In Situ Optical Spectroscopy

**DOI:** 10.1021/acsami.2c09496

**Published:** 2022-07-27

**Authors:** Angela Terracina, Angelo Armano, Manuela Meloni, Annamaria Panniello, Gianluca Minervini, Antonino Madonia, Marco Cannas, Marinella Striccoli, Luca Malfatti, Fabrizio Messina

**Affiliations:** † Dipartimento di Fisica e Chimica, Università degli Studi di Palermo, Via Archirafi 36, 90123 Palermo, Italy; ‡ Department of Chemistry and Pharmacy, Laboratory of Materials Science and Nanotechnology, CR-INSTM, University of Sassari, Via Vienna 2, 07100 Sassari, Italy; § CNR-IPCF-Bari Division, c/o Chemistry Department, and Chemistry Department, University of Bari “Aldo Moro”, Via E. Orabona 4, 70126 Bari, Italy; ∥ Department of Electrical and Information Engineering, Polytechnic of Bari, Via E. Orabona, 4, 70126 Bari, Italy

**Keywords:** carbon nanodots, photobleaching, photoresistance, fluorescent nanoparticles, diffusion, time-resolved dynamics

## Abstract

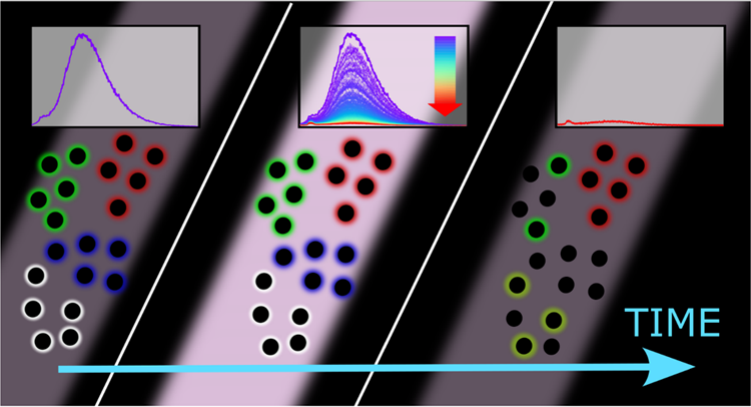

Carbon dots (CDs) are a family of fluorescent nanoparticles displaying a wide range of interesting properties, which make them attractive for potential applications in different fields like bioimaging, photocatalysis, and many others. However, despite many years of dedicated studies, wide variations exist in the literature concerning the reported photostability of CDs, and even the photoluminescence mechanism is still unclear. Furthermore, an increasing number of recent studies have highlighted the photobleaching (PB) of CDs under intense UV or visible light beams. PB phenomena need to be fully addressed to optimize practical uses of CDs and can also provide information on the fundamental mechanism underlying their fluorescence. Moreover, the lack of systematic studies comparing several types of CDs displaying different fluorescence properties represents another gap in the literature. In this study, we explored the optical properties of a full palette of CDs displaying a range from blue to red emissions, synthesized using different routes and varying precursors. We investigated the photostability of different CDs by observing in situ their time-resolved fluorescence degradation or optical absorption changes under equivalent experimental conditions and laser irradiation. The results about different PB kinetics clearly indicate that even CDs showing comparable emission properties may exhibit radically different resistances to PB, suggesting systematic connections between the resistance to PB, the characteristic spectral range of emission, and CD quantum yields. To exploit the PB dynamics as a powerful tool to investigate CD photophysics, we also carried out dedicated experiments in a partial illumination geometry, allowing us to analyze the recovery of the fluorescence due to diffusion. Based on the experimental results, we conclude that the nature of the CD fluorescence cannot be solely ascribable to small optically active molecules free diffusing in solution, contributing to shed light on one of the most debated issues in the photophysics of CDs.

## Introduction

Since their discovery in 2004,^1^ carbon dots (or CDs) have been attracting increasing interest because of their large range of remarkable properties and possible uses,^2−4^ as well as their ease of fabrication with a manifold of different techniques like electrochemical method,^5^ microwave method,^6^ laser etching,^7^ solvothermal method,^8,9^ and so on. In particular, thanks to their tunable fluorescence and favorable photochemistry, these emerging zero-dimensional fluorescent nanoparticles have shown great potential in bioimaging,^10^ photocatalysis,^11^ optoelectronics,^12^ optical sensing,^13^ photovoltaic applications^14^ and so on. CDs are nanometer-sized particles exhibiting bright fluorescence and usually structured as quasi-spherical carbonaceous cores covered by a dense shell of chemical functional groups.^15,16^ Beside carbon, CDs typically contain significant amounts of oxygen and nitrogen, especially in the surface shell, and high contents of nitrogen in particular have been connected to high emission quantum yields.^17,18^ Anyway, the characteristics of CDs strongly depend on the precursors employed for the synthesis, which may involve organic molecules or polymers containing, for example, hydroxyl, carboxyl, amides, or amino groups,^19−21^ which in turn leads to a wide diversity of structural and optical properties. Because of this, despite more than 15 years of dedicated studies, the fundamental photoluminescence mechanism is still one of the most unclear characteristics of CDs and wide differences exist in the literature concerning the reported optical response and photostability.^17,22^ The usually intense and tunable fluorescence, which is often very sensitive to local environments (e.g., solvents or pH),^15,23^ has been attributed sometimes to the formation of molecular fluorophores attached to the CDs surface, to energy traps located at the surface of the carbon cores, to intrinsic emissions arising from the quantum confinement effect, and also to an interplay between these intrinsic and extrinsic states.^15,17,24−27^ Besides a poor understanding and control of their optical properties, another crucial issue in the field is the difficulty to obtain bright red-emitting CDs,^28−31^ as opposed to the strong absorption and high quantum yields often achieved for blue- and green-emitting CDs. A further serious complication is the frequent presence of side products of CD synthesis consisting of free emitting small molecules, which may be hard to separate from CDs. Indeed, these molecules may give their contribution to the total observed fluorescence, making it very tricky to isolate the intrinsic contribution arising from CDs only.^22,32−38^

Another challenging aspect currently hampering CD applications concerns their photostability: in fact, despite previous claims of high photostability,^24,39,40^ many recent works have highlighted the occurrence of photobleaching (PB) effects under prolonged illuminations.^41−44^ This aspect constitutes a strong limit because it leads to their degradation and also because it may generate blinking and bleaching effects in the single dots^22,45,46^ that are detrimental for bioimaging applications. Furthermore, the photodegraded CDs may be harmful to health,^47^ thus compromising one of the most important features of CDs compared to semiconductor quantum dots, which is their nontoxicity and biocompatibility.^48−50^ However, it has also been found that PB may be useful to investigate and even control the optical properties of CDs.^22,42−44,51^ For example, Rogach and co-authors^52^ have recently observed that, by taking advantage of the significant difference in photostability found between the emission arising from the intrinsic state and that arising from the surface molecular fluorophores, the former contribution can be isolated upon a prolonged UV irradiation, thus obtaining a narrow and bright intrinsic photoluminescence. Instead, other studies have focused on devising methods to enhance photostability.^46^ Recently, a possible relation between quantum yield (QY) and sensitivity to PB has also been hypothesized, but only UV radiation was used as a bleaching source in that work, as in the large majority of the existing studies.^22,44,53^ Indeed, only a few studies have addressed PB of CDs under strong visible light beams^54^ and tightly controlled conditions (e.g., laser irradiation), despite the direct relevance for applications, and most studies have only observed the net effect of the PB process, without investigating the kinetics in real time.^44^ Overall, further efforts are needed to systematically correlate the resistance to PB to the variable characteristics of CDs ranging from blue to red emitters, and to find a predictive relation between photostability and their structural and optical features.

The present work aims to bridge the current gaps in the study of these problems. We have used time-resolved optical measurements in situ at the irradiation site to achieve a real-time reconstruction of PB effects and their kinetics. We repeated these experiments on a wide palette of CDs synthesized from different precursors in such a way to display emissions ranging from blue to red. Thereby, we have identified some general trends or discrepancies concerning the photostability among CDs with different or similar emission features, providing hints to predict the degree of photostability of a determined CD type. In particular, our findings pointed out that even CDs displaying almost identical fluorescence may have very different resistances to PB, while a certain correlation between resistance to PB and fluorescence lifetime has been observed. Finally, by changing illumination geometry between total and partial illumination, we were able to estimate the size of the small molecular units contributing to CD emission, finding that part of them is freely diffusing in solution. However, in this way, we have also demonstrated that, in the explored palette of CDs, these molecules actually contribute to the observed fluorescence only to a relatively small extent. Our findings strongly contribute to clarify CD photobleaching pathways and to provide important information on their fundamental fluorescence mechanisms. Our results provide a step forward toward antifading CDs in view of their industrialization in real-world applications.

## Materials and Methods

### Synthesis of Carbon Dots

We studied a heterogeneous palette of CDs produced by a variety of different methods and displaying fluorescence in different regions of the visible spectrum. Their PB dynamics were studied in an aqueous solution, where these CDs are all highly dispersible due to their hydrophilic surface groups. The samples are named CT, CU2 (blue emitters), CZAU and CU25 (green emitters), and SAFD (red emitters). To further extend the scope of the study, we also considered an additional type of CD, named CD49, which has a hydrophobic character. Experiments on CD49 were conducted in chloroform. The detailed preparation and purification procedures of these samples are described hereafter.

#### Materials

Citric acid (CA, Fluka, purity > 99.5%), 2-amino-2-(hydroxymethyl)propane-1,3-diol (Tris, Carlo Erba, purity > 99.5%), urea for electrophoresis (U, Sigma-Aldrich, purity 98%), citrazinic acid (CZAc, Alpha Aesar, purity 97%), Safranin O dye (Sigma-Aldrich), hydrochloric acid (Sigma-Aldrich, 37% wt/wt), water (milli-Q), Octadecene, ODE (Sigma-Aldrich, 90%), 1-hexadecylamine, and HDA (Fluka, 98%) were used as received without further purification. Solvents (acetone, chloroform, ethanol) were of analytical grade and purchased from Aldrich or Fluka.

#### Synthesis of CT

The synthesis was performed according to a previous work.^55^ Citric acid monohydrate (CA, 1261 mg, 6.0 mmol) and Tris (242 mg, 2.0 mmol) were directly placed in a round-bottom flask in their crystalline form and immersed in a preheated 180 °C oil bath (100 rpm stirring rate was applied with a Teflon coated stirring bar). During the reaction, the reagents melt to form a transparent liquid that slowly turned light brown. After the thermal treatment, the products were dissolved in water and filtered with a 0.22 μm syringe filter.

#### Synthesis of CU2, CU25, and CZAU

Citric acid-urea dots (CU2 and CU25) were synthesized according to previous work,^56^ and the same protocol was also adapted for CZAU. Briefly, we dissolved in three stocks of water (10 mL), 0.96 g of CA and 1.2 g of U (molar ratios of 1:2, CU2); 0.19 g of CA and 1.5 g of U (molar ratio 1:25, CU25); and 0.15 g of CZAc and 1.5 g of U (molar ratio 1:25 CZAU). After dissolution of the reagents, the solutions were heated in an open vessel using an oil bath at 190 °C for 2 h. After thermal degradation, the final products appeared as a black solid powder. The powders were dissolved in water and centrifuged at 5000 rpm, for 10 min, to remove the larger aggregates. Then, the supernatants were filtered with a 0.22 μm syringe filter. Solutions at a concentration of 0.01 mg mL^–1^ were prepared for the UV–vis spectroscopy and photoluminescence (PL) analysis.

#### Synthesis of SAFD

CA (3.84 g, 0.02 mol) was added into a 50 mL round flask in its crystalline form and dipped into a preheated 200 °C oil bath. Afterward, 0.35 g of Safranin O (SO, 0.001 mol) was added when CA was completed melted. After 2.5 minutes of reaction time, the obtained thermal product results in a dark-red precipitate. The precipitate was dispersed in Milli-Q water and purified through dialysis bags (cutoff ∼14,000 Da). The resulting SAFD solution was then dried at 60 °C in an oven to obtain the solid powders. (submitted to Nanomaterials)

#### Synthesis of CD49

It was carried out by a previously reported procedure,^28^ involving thermal carbonization of CA as carbon source in the presence of hexadecylamine (HDA) in a high-boiling-point solvent (octadecene, ODE), under nitrogen atmosphere. Specifically, 6 mmol of HDA was dissolved in 15 mL of ODE and degassed under vacuum at 110 °C for 30 min. Then, the mixture was heated up to 200 °C under nitrogen flux and 5 mmol of anhydrous CA were quickly added to the reaction batch, under vigorous stirring. The heating was kept at 200 °C for 3 h, under nitrogen flux, and finally, the reaction was quenched by cooling down at room temperature. C-dots were isolated and purified from the reaction batch by numerous cycles of washing with acetone and precipitation steps by centrifugation at 10,000 rpm. The resulting hydrophobic C-dots were finally dispersed in chloroform.

### Transmission Electron Microscopy (TEM)

TEM analysis was performed using an FEI Tecnai 200 microscope working with a field emission electron gun operating at 200 kV and a JEOL JEM1011 microscope, equipped with a W filament operating at 100 kV, and acquiring the images by an Olympus Quemesa CCD camera. Samples were prepared by depositing a properly diluted CD dispersion onto carbon-coated copper grids. Statistical analyses were conducted using a free image analysis software (ImageJ, v.1.52a) to gain information on CD average size and size distribution.

### Attenuated Total Reflection (ATR)

ATR spectra were measured using a Bruker Platinum ATR spectrometer equipped with a single-reflection diamond crystal in the range 400–4000 cm^–1^. A single drop of sample solution was deposited onto the sample holder and let to dry before the measurement.

### Steady-State Optical Absorption

Optical absorption (OA) spectra of CD solutions were acquired in a 1 cm quartz cuvette by an optical fiber spectrometer (Avantes AvaSpec-uls2048cl-evo-rs) in the range of 200–1300 nm.

### Steady-State Photoluminescence

Steady-state photoluminescence (PL) spectra of CD solutions were acquired by a spectrofluorometer (Jasco FP6500) equipped with a 150 W Xe lamp as a light source. Excitation and emission wavelengths varied between 220–550 and 230–700 nm, respectively.

### Time-Resolved Photoluminescence

Time-resolved photoluminescence (TRPL) of CDs was investigated using the following setup: a monochromator (Spectra Pro2300i PI Acton), a time-gated CCD camera (PI-MAX), and a pulsed laser (Opotex Vibrant) with 10 Hz pulse frequency, 5 ns pulse width. Laser and CCD were synchronized to acquire PL spectra as a function of time delay after excitation of CDs by the laser pulse. PL lifetimes were extracted by least-squares fitting the temporal kinetics of PL intensity.

### Quantum Yield (QY)

The emission efficiency of CDs was evaluated by the estimation of their quantum yield by comparison with fluorescent standards having a similar emission range: fluorescein sodium salt (purchased by Sigma-Aldrich) dispersed in water at pH = 12 for green-emitting CDs and Coumarin-2 (purchased by Lambda Physik) diluted in water for blue-emitting CDs. Relative QY measurements were carried out using the same geometry for the sample and the reference dye. The absolute QY of the reference dyes was measured using a general-purpose integrating sphere (Labsphere 3p-gps-060sf, ig) internally covered by Spectralon, obtaining the following values: QY = (62 ± 4)% for fluorescein and QY = (94 ± 9)% for coumarin-2. The QY of SAFD was directly obtained using the integrating sphere, as described for the reference dyes. For comparison purposes, we also studied a sample of CZAc dispersed in water, whose QY was obtained by comparison with Coumarin-2 dye, similarly to blue-emitting CDs.

### Photobleaching

Fluorescence loss in photobleaching (FLIP) experiments were performed by exposing CD samples to an alternating sequence of bleaching and probing laser pulses, or to pulses from a single laser serving both as bleaching and probing beam, allowing us, in both cases, to monitor the evolution of CD fluorescence intensity as a function of the increasing number of administered bleaching pulses. The fluorescence PB was investigated in the range of 0–10^5^ pulses (about 3 h, using a repetition rate of 10 Hz). Time-dependent fluorescence intensity was estimated by averaging spectra excited by 10 consecutive probe pulses. All of the experiments were performed using 40 μL of the sample solution in a 1 mm quartz cuvette. The geometry was chosen in such a way to ensure that the whole volume was irradiated by the bleaching beam.

**Figure 1:**
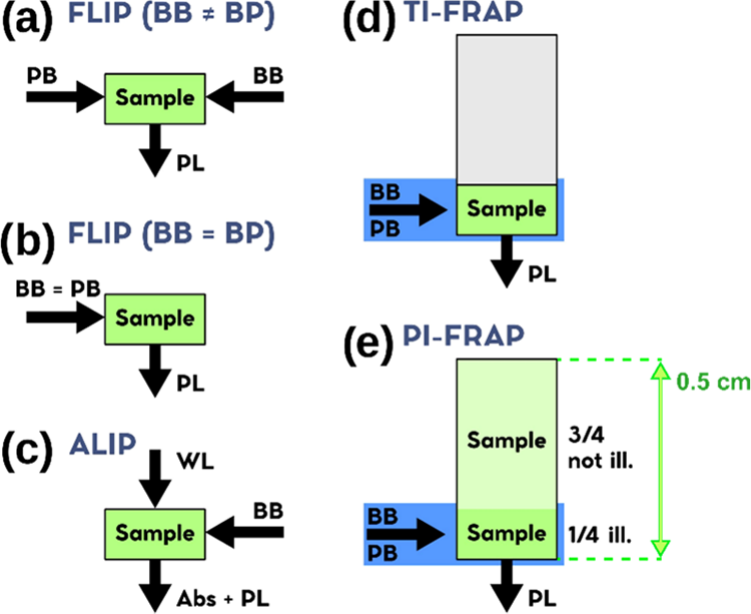
Schemes of different PB experiments: (a) fluorescence loss in photobleaching (FLIP) using distinct or (b) coincident probe (PB) and bleach (BB) laser beams, and the photoluminescence intensity (PL) as an observable; the wavelength of both probe and bleach beams is chosen to be in resonance to the main absorption features of the samples, that is, λ_pump_ = 355 nm, λ_probe_ = 320–360 nm (blue-emitting CDs), λ_pump_ = λ_probe_ = 400–430 nm (green-emitting CDs) and λ_pump_ = λ_probe_ = 532 nm (red-emitting CDs). (c) Absorption loss in photobleaching (ALIP). Here, a pulsed laser beam is used to bleach the PL activity of the CDs, while a continuous white light beam is used to simultaneously probe (WL) the absorbance (Abs); (d) total illumination (TI) and (e) partial illumination (PI) geometries used for fluorescence recovery after photobleaching (FRAP) experiments. In all of the experiments, the bleaching pulse energy was always fixed at 1.0 mJ, with a beam diameter of 1 mm, corresponding to about 130 mJ/cm^2^ fluence per pulse.

In the case of blue CDs, two different laser beams with different intensities were used for probing and bleaching (Figure 1a). The third harmonic of a Q-switched Nd:YAG pulsed laser (Quanta System SYL-201) with 10 Hz pulse frequency, 5 ns pulse width, 1.0 mJ pulse energy, and 355 nm wavelength was used as bleaching beam. The probe laser beam, with the same wavelength but lower pulse energy (0.04 mJ) was obtained from a pulsed tunable laser (Opotex Vibrant) with 10 Hz pulse frequency and 5 ns pulse width. The 355 nm wavelength of both beams matched the longest-wavelength absorption band of our CU2 and CT blue-emitting samples. The same experimental conditions have been used for comparison experiments conducted on the CZAc sample and on the hydrophobic CD49 sample.

In the case of green CDs, we probed the effects of PB by monitoring the fluorescence excited by the bleaching beam itself, thus allowing the use of a single beam for both bleaching and probing (Figure 1b). The beam was obtained through the same tunable laser described above (Opotex Vibrant), using a 1.0 mJ pulse energy and a wavelength tuned in resonance with the significant absorption features of samples CZAU, CU25, and CD49, typically in the 410–430 nm range.

We used the single-laser configuration also for red-emitting CDs, using a single-pulsed beam at 532 nm, 1.0 mJ/pulse, as both the bleaching and probing source (Figure 1b). For the sake of comparison, we also carried out an additional FLIP experiment on red-emitting CDs where we used a bleach beam at 266 nm and a probe beam at 532 nm.

Absorption loss in photobleaching (ALIP) experiments were performed by exposing the samples to a pulsed bleach beam having the same characteristics discussed above, while monitoring their absorption spectra through a white probe beam provided by a discharge lamp. Thereby, we acquired the evolving absorption spectra of CDs under 1.0 mJ bleaching pulses (Figure 1c). The absorption decay was investigated in the range (0–1) × 10^5^ pulses (about 3 h). Each absorption spectrum was averaged over 50 bleaching pulses. All of the experiments were performed using 80 μL of sample solution in a 0.5 cm quartz cuvette. The geometry was chosen in such a way to ensure that the whole volume was irradiated by the bleaching beam.

Finally, in fluorescence recovery after photobleaching (FRAP) experiments, CD samples were first subjected to a bleaching stage by administering 2 × 10^4^ bleach pulses at the same experimental conditions used for the analogous FLIP experiment, which are generally found to bleach more than 80% of fluorescence. Thereafter, we stopped the bleaching beam and used the probe beam to reveal possible recovery phenomena of the fluorescence over time, after the end of the bleaching phase. Two different sample illumination configurations were investigated in FRAP experiments: total illumination (Figure 1d, TI-FRAP) and partial illumination (Figure 1e, PI-FRAP). While in TI-FRAP the bleaching beam invested the entire volume of the sample, in PI-FRAP, both bleaching and successive probing experiments were conducted in a region of interest consisting of about 25% of the total sample volume contained in the cuvette. Simulations: Computer simulations of diffusion phenomena were performed by Energy2D, which is a free software that allows us to simulate heat transport in two-dimensional geometry,^57^ taking advantage of the formal coincidence between the heat transport and diffusion partial differential equations.

## Results and Discussion

### Preliminary Characterization of the Samples

The CDs investigated in this study were produced by a range of bottom-up synthesis routes based on the thermal decomposition of molecular precursors (see the Materials and Methods section for details). Typically, such kind of bottom-up synthesis allows fine tailoring of the structure and properties of CDs due to the multiple process parameters (temperature, time, pressure, and solvent) and an endless variety of carbonaceous reagents that can be used. Typically, the precursors are an organic acid, used as the main carbon source, combined with an amine or amide as a nitrogen source. Suitable choices of the precursors and reaction conditions enable an effective tuning of the emission properties across the whole visible spectrum. The syntheses were carefully selected to obtain a whole set of CDs emitting in the blue, green, and red ranges of the visible spectrum.

Our CD samples are grouped into three families based on their fluorescence color and named: CT, CU2 (blue emitters), CZAU and CU25 (green emitters), SAFD (red emitters). All of these samples are highly dispersible in water and other polar solvents. We also included a fourth type of CDs, named CD49, which is hydrophobic and displays a blue-green dual emission.

The formation of CDs was confirmed by transmission electron microscopy (TEM) imaging, as shown in Figure S1. TEM analysis of CZAU (Figure S1c) reveals carbon dots with a size distribution between 15 and 25 nm, whereas analysis of CT (Figure S1e) and SAFD (Figure S1d) shows smaller sizes in the 1–10 nm range. Analysis of CD49 reveals an average size of about 2 nm (Figure S1f). TEM analysis on CU2 and CU25 (Figure S1a,b) reveals the formation of larger particles with sizes in the range of 10 to ∼35 nm.

**Figure 2:**
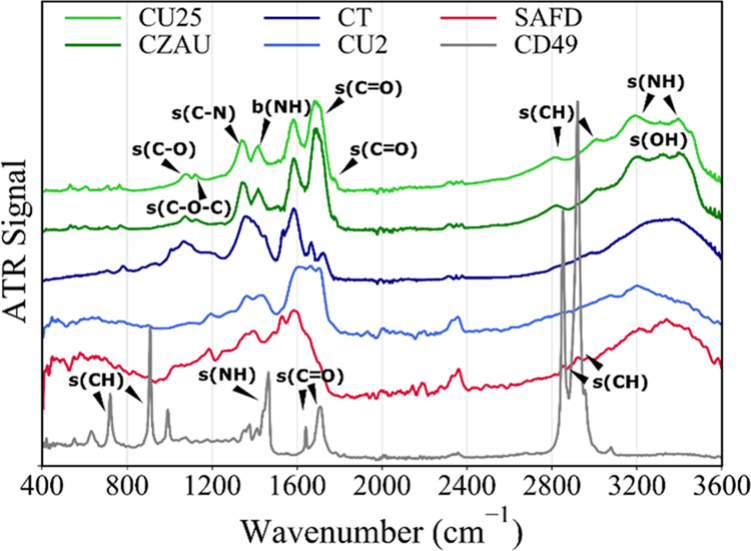
Attenuated total reflectance (ATR) infrared absorption spectra of CD samples. From top: CU25, CZAU, CT, CU2, SAFD, and CD49. The labels highlight the attributions of some of the prominent signals observed in the spectra.

Then, the surface chemistry of CDs was investigated by vibrational spectroscopy. As shown by attenuated total reflectance (ATR) spectra in Figure 2, all samples except CD49 display a large degree of similarity, and especially CZAU and CU25 are almost indistinguishable, even in the fingerprint region at lowest wavenumbers. The surface of all CDs is rich in C=O, C–OH, and −NH_2_ functional groups, stemming from the functional groups present in the original precursors. From Figure 2, all of the specimens show the broad band at 3200–3300 cm^–1^ due to OH stretching, while the minor contributions at ≈2800 and ≈3000 cm^–1^ can be attributed to CH stretching. The narrower features at ≈3200, 3400, and 3450 cm^–1^ that we attribute to NH stretching are only observed in CU25 and CZAU. A broad C=O stretching band^58^ at 1690 cm^–1^ and a weaker contribution at 1710 cm^–1^ are prominent in the spectra of CU25 and CZAU, but C=O vibrations in this region also appear in lower intensity in the CT sample and can be barely discerned in the CU sample. At lower wavenumbers, we found a third broad band at 1580 cm^–1^ and signals at 1345 and 1415 cm^–1^, which can be respectively attributed to C–N stretching and N–H bending in amines, confirming successful N-doping of the CD surface.^58^ Again, these N-related signals are clearly distinguishable in CU25 and CZAU but less resolved in CT and CU2, where the concentration of N in the original precursors is much lower. Finally, a poorly structured band is found in the range 1000–1100 cm^–1^, likely ascribable to C–OH stretching. Despite CU2 and CU25 having the same precursors, although in different proportions, their ATR spectra are quite different: in particular, the CU2 spectrum is poorly resolved, but it is possible to recognize the C=O features and the peaks involving C–N and N–H stretching. SAFD spectrum is equally unresolved: its main peaks are at ≈1530 and 1570 cm^–1^, likely arising from the nitroaromatic rings.^58^ Vice versa, the hydrophobic sample CD49 shows very sharp and strong IR peaks in the spectral region at 600–1000 cm^–1^ and at 2800–3000 cm^–1^. Such peaks are ascribable to the C–H stretching of long alkyl chains deriving from aliphatic compounds employed for CD synthesis, including also residual high-boiling ODE and HDA (see the Materials and Methods section), that form the hydrophobic surface shell of the CDs.^28^ In particular, the sharp signal at 1465 cm^–1^ is ascribable to N–H stretching of amine groups of HDA. The CD49 sample also shows a C=O stretching peak at 1705–15 cm^–1^, which can be attributed to the formation of amide groups (−CONH_2_) through the condensation reaction of CA and the primary amine groups of HDA.^59^ Finally, the weaker and sharper signal at 1640 cm^–1^ can be attributed to the C=O stretching of carboxyl groups in CDs.

**Figure 3:**
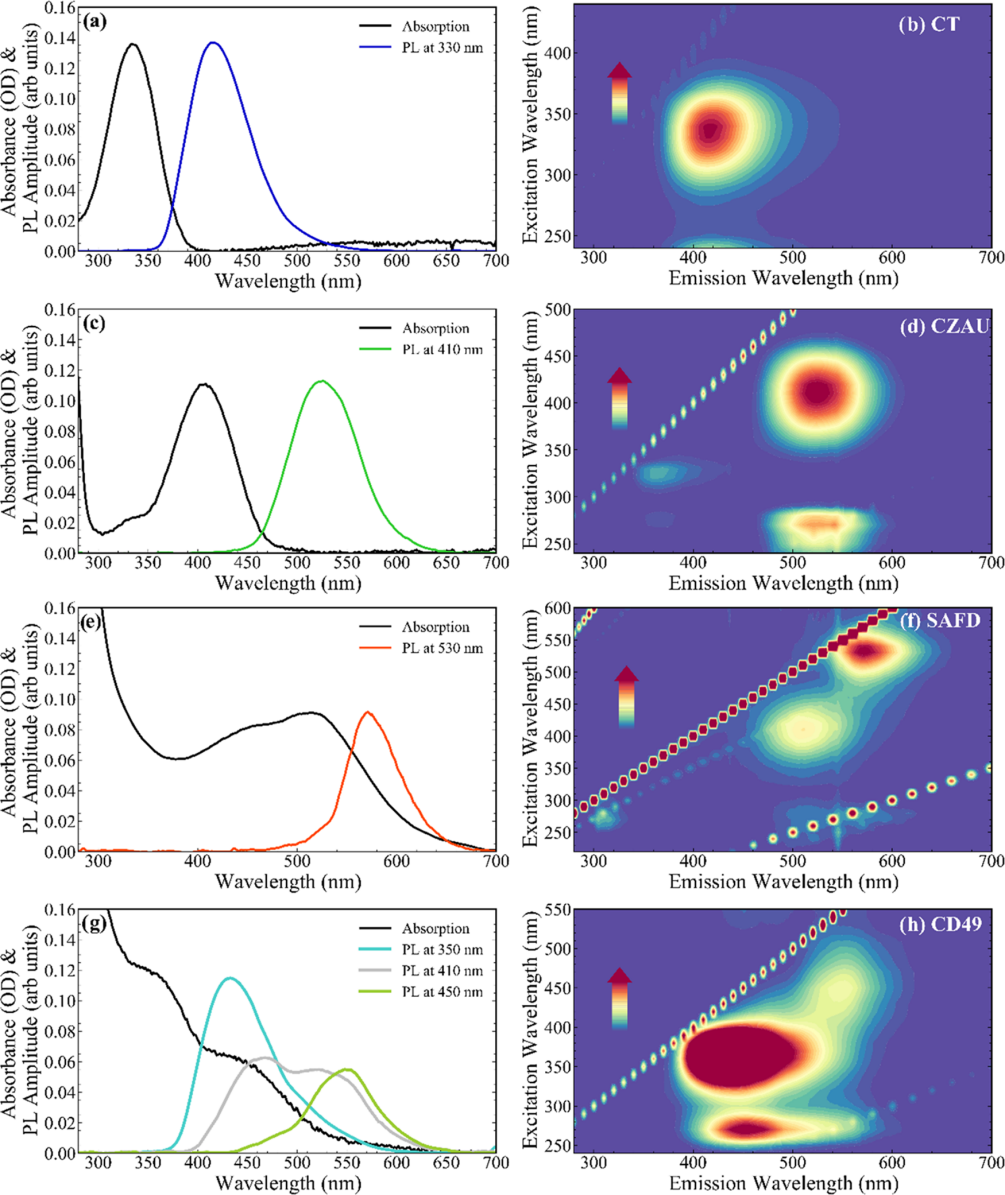
Optical absorption of representative hydrophilic CD samples emitting in the blue (CT), green (CZAU), and red (SAFD) regions (a, c, e, respectively) and of the hydrophobic CD49 sample having a dual emission (g). The absorption spectra are shown together with PL spectra collected exciting in correspondence of the absorption maxima. The two-dimensional excitation–emission fluorescence intensity maps of the same samples (b, d, f, h) allow us to understand the nontunable character of the samples’ emissions and their mirror symmetry with the corresponding excitations.

After the structural characterization, the native optical properties of CD samples were deeply characterized as a starting point to better understand the PB effects. The optical absorption (OA) spectra of all CD samples are reported in Figure S2: all of the spectra have in common a strong absorption in the deep-UV region (λ < 300 nm), due to the onset of π–π* transitions of sp^2^ C bonds in the carbonaceous cores.^60−62^ At lower energies, we observe further transitions, which are found to be strongly sample-dependent. These bands are the most interesting features in each spectrum since they are directly related to fluorescence. A more detailed view, for representative CDs (CT, CZAU, SAFD, and CD49) belonging to the four sample groups defined above, is shown in Figure 3 along with the correspondent fluorescence spectra excited at their characteristic absorption wavelengths, whereas the analogous spectra of the remaining samples are shown in Figure S3.

Samples CT, CZAU, and SAFD show a prominent lowest-energy absorption transition at 330, 410, and 520 nm, respectively. Emission spectra recorded exciting in correspondence of these absorption maxima, showing fluorescence bands approximately mirror-symmetric to the absorption bands, peaking in the blue (≈410 nm), green (≈520 nm), and orange (≈570 nm) regions, respectively. Such fluorescence bands are characterized by a pronounced Stokes shift of about ≈80, 110, and 50 nm from the absorption peaks, respectively. In some cases, additional minor contributions to the overall fluorescence can be discerned, such as an additional green emission in the SAFD sample (λ_exc_ ≈ 410 nm, λ_em_ ≈ 510 nm; see the two-dimensional (2D) map in Figure 3f). Conversely, the CD49 sample displays two distinct absorption bands at 360 and 450 nm, and likewise two fluorescence bands having a Stokes shift of ≈90 nm, hence occurring in the blue-green (λ_exc_ ≈ 360 nm, λ_em_ ≈ 430 nm) and green-yellow regions, respectively. Such fluorescence bands, if properly combined by opportune excitation selection, can be exploited to obtain a white emission, as reported before.^28^

Similar investigations were performed also for samples CU2 and CU25 (Figure S3), highlighting similar features. The OA of the blue-emitting CU2 sample is broader than the other blue-emitting CT due to the presence of an additional shoulder around 450 nm (compare Figure 3a with Figure S3a), but it still displays a well-defined peak at about 350 nm, which excites a comparatively narrow fluorescence band. Concerning the green-emitting samples, the optical properties of CU25 and CZAU are very close to each other, both displaying a well-defined absorption peak at ≈410 nm with a very similar shape, which excites green fluorescence peaked at ≈520 nm.

The optical data in Figure 3 confirm that the choice of the precursors and synthesis conditions have a deep effect on the fluorescence response of CDs. Interestingly, a common characteristic of almost all of the samples considered in this work is the almost complete lack of fluorescence tunability with excitation wavelength (see 2D fluorescence maps in Figures 3 and S3), which contrasts with the frequent observation of a strong excitation dependence of the emission peak of CDs.^10,15,23,63^ Several recent studies have shown that the fluorescence tunability of CDs is ultimately connected to the heterogeneity of surface states and charge traps^62,64^ or to a cocktail of different fluorophores contributing to the fluorescence of a given CD sample.^25,65^ In contrast, the nontunable fluorescence observed here and the approximate absorption–emission mirror symmetry are typical molecular-like behaviors ultimately stemming from Kasha’s rule. Therefore, it seems reasonable to suppose that all of these CDs emit through specific electronic transitions of molecular fluorophores formed during the synthesis. In fact, a significant role of molecular units in the fluorescence of carbon dots is now well established,^18^ at least for CDs produced by bottom-up methods.^34^ Here, the fluorescence bands of the blue-emitting CDs (CT and CU2, Figures 3 and S3, respectively) are very close to that of CZAc (vide infra). On the other hand, those of green CDs (CU25 and CZAU) may resemble HPPT (4-hydroxy-1*H*-pyrrolo[3,4-*c*]pyridine-1,3,6(2*H*,5*H*)-trione).^18,35^ Both these molecules have been previously found as reaction side products of typical CD bottom-up synthesis routes.^18,35^ In the sense of SAFD, the fluorescent unit is expected to be closely related to Safranin O, which is used as a precursor. However, based on these data alone, it is not possible to easily discriminate freely diffusing fluorescent molecules from molecular fluorophores effectively embedded in the structure of CDs or adsorbed on CDs surfaces, and thus affected by the interactions with the CD cores.

To complete the optical characterization, the excited-state dynamics and emission efficiency of CDs were evaluated by quantum yield (QY) measurements and time-resolved photoluminescence (TRPL). The time-resolved fluorescence data are shown in Figure S4, while Table S1 summarizes the results of TRPL and QY studies. We found that the QY systematically increases going from red-emitting to blue-emitting CDs. SAFD displays the lowest QY value of 4%, followed by CU25 and CZAU, showing QY values between 10 and 20%, and finally by blue-emitting CDs, showing systematically higher QYs: 57% for CT and 32% for CU2. The hydrophobic CD49 sample also follows the same trend, displaying QY = 46% for the blue emissive component (named CD49B) excited at 370 nm, and QY = 7% for the green-yellow component (CD49G) excited at 450 nm. Time decay of CD samples measured by TRPL (see Figure S4 and Table S1) shows that blue CDs are characterized by the longest lifetimes (τ = 7.7–9.5 ns), whereas green CDs show shorter lifetimes (τ = 2.6–6.8 ns), further decreasing in the red SAFD sample (1.3 ns).

Interestingly, most of the TRPL data cannot be reproduced by single-exponential decays. All of the decay curves are indeed fitted by a stretched-exponential law *I*(*t*) = *I*_0_ exp(−(*t*/τ)^β^). This law is frequently used as a phenomenological model to represent the dynamic behavior of a disordered system displaying a statistical distribution of decay lifetimes. In particular, the parameter τ represents the average decay lifetime, while the degree of deviation of the parameter β from 1 (0 < β < 1) provides an estimate of the degree of disorder. This type of decay kinetics is not consistent with simple molecular fluorophores in the solution phase and directly suggests that the molecular fluorophores responsible for CD emissions experience a certain degree of heterogeneity in their local environments. Notable differences in stretching factors β are obtained among the CD samples: from Table S1, the highest degrees of heterogeneity (β significantly below 1) are observed for CT, for green CDs, and for the blue component of CD49.

Despite the lack of fluorescence tunability highlighted by steady-state fluorescence measurements, the heterogeneity indicated by the stretched-exponential decays might be indicative of significant interactions of the emissive molecular unit with the carbonaceous CD cores. For example, despite the very close resemblance of CU2 emission band shape to that of free CZAc (data in Table S2 and Figure S5), we measured a higher QY (32% against 19% of CZAc) and a longer lifetime (8.9 ns against 6.0 ns for CZAc). Besides, the fluorescence decay curve for the free molecular dye can be perfectly fitted by a single-exponential function (see fit in Figure S5). Similarly, we found that although the emission band shape of red SAFD is fairly close to the precursor Safranin O used in the synthesis (not shown), SAFD QY is lower (4% against 16% measured for pure Safranin O). Overall, our data suggest that the picture of CD emission as trivially arising entirely from a mixture of free diffusing molecular fluorophores^36,38^ is not appropriate here. As shown in the next sections, PB experiments provide an alternative route to address these problems allowing, among other things, to discriminate freely diffusing molecules from carbon dots.

### Photobleaching of CDs

We carried out a series of PB experiments aiming to evaluate the differences in photostability among CD samples and also as a way to obtain further information about the nature of the fluorophores responsible for CD emission.

**Figure 4:**
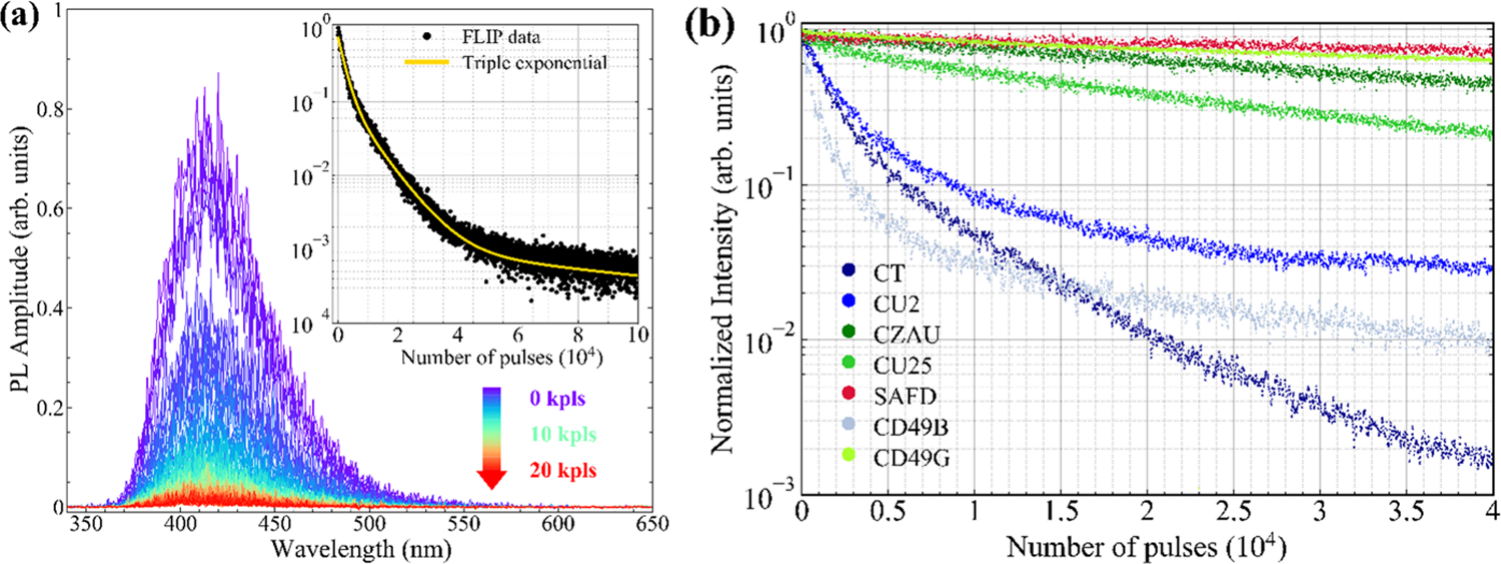
(a) Fluorescence loss in photobleaching (FLIP) experiment on CT sample. The color scale from blue to red indicates the increase in the number of bleach pulses (355 nm) received by the sample (shown only up to 20,000 pulses in steps of 250 for sake of clarity). The decay of the fluorescence intensity (integrated in the range from 350 to 600 nm, and normalized to the initial intensity) is shown in the inset. The resulting curve of triple exponential fit is also reported. (b) Comparison of FLIP decay curves obtained for all CD samples. The excitation wavelengths used for each sample are listed in Table S3.

The effect of intense laser illumination on CDs was first investigated through fluorescence loss in photobleaching (FLIP) experiments, where the temporal evolution of CD fluorescence is monitored while the samples are exposed to a series of intense bleach pulses (details in the Materials and Methods section). The results obtained for the CT sample are shown in Figure 4a as an illustrative example. Other representative samples are shown in Figure S6. In Figure 4a, the experiment was conducted by irradiating the sample with 1 mJ laser pulses (5 ns) at 355 nm, a wavelength close to the excitation peak of the blue fluorescence transition. As shown, the fluorescence of CT progressively fades with increasing the number of bleach pulses received by the sample, clearly indicating the destructive effect of intense illumination on the emission properties of the CD sample. In fact, the fluorescence band almost completely disappears after about 10^5^ laser pulses.

The decay of CT fluorescence is summarized in the inset graph in Figure 4a, where the intensity is plotted on a semi-log scale as a function of the number of bleach pulses received. A clear non-single-exponential dependence is found, suggesting that more than one process is involved. More in detail, the decay of the emission intensity *I*(*N*) can only be satisfactorily fitted by a three-exponential function: *I*(*N*) = *A*_1_ exp(−*N*/*N*_1_) + *A*_2_ exp(−*N*/*N*_2_) + *A*_3_ exp(−*N*/*N*_3_), with the fitting parameters reported in Table S3. For the sake of simplicity, hereafter, the overall efficiency of PB will be synthetically expressed by the parameter *N*_1/2_, which is the number of pulses needed to halve the fluorescence intensity. For CT sample, we obtained *N*_1/2_ = 1.2 × 10^3^ pulses. A similar experiment was performed for each sample, exposing it to 1 mJ laser pulses at the respective peak excitation wavelength of the fluorescence. As a result, we obtained the FLIP curves shown in Figure 4b. The corresponding fitting results are summarized in Table S3. We find that PB is a ubiquitous phenomenon for all of the investigated CDs. All of the FLIP kinetics are markedly non-single-exponential, with the only exception of CZAU and SAFD, two of the CDs displaying the slowest PB.

Interestingly, no significant changes in the shape of the different fluorescence bands are observed during the progress of PB (see Figure S6). Once again, this result strongly suggests that the fluorescence may be attributed to a specific fluorophore that, considering the strong nonexponential character of the PB, experiences different local interactions with CD cores, able to limit, more or less effectively, the PB effects. This picture contrasts with previous findings reporting that PB can be used to eliminate the contribution of molecular fluorophores to the overall emission,^52^ so allowing us to isolate fluorescence related to core states. For our CDs, the fundamental fluorescence features do not undergo significant changes albeit a strong intensity reduction. Thus, the fluorescent unit is only one for each of the CD samples, except in the case of CD49 where two different fluorescence bands (blue and green) coexist and have different photoresistances. Based on previous work,^28^ the blue band can be attributed to superficial states, whereas the green-yellow band can be ascribed to molecular fluorophore.

Despite a strong heterogeneity in the resistance to PB among samples, some common trends appear. On the one hand, the photoresistance clearly increases from blue- to red-emitting CDs. The former CDs show the lowest photostability, typically losing 50% of the initial fluorescence within 2000 laser pulses or less. For the blue emitters in our palette of CDs, we obtained: *N*_1/2_ = 0.4 × 10^3^ pulses for CD49 (blue emissive component), *N*_1/2_ = 1.2 × 10^3^ pulses for CT, and 1.3 × 10^3^ pulses for CU2. In contrast, green CDs are much more photostable, with half-times of *N*_1/2_ = 80 × 10^3^ pulses (CD49, green component), *N*_1/2_ = 40 × 10^3^ pulses (CZAU), and *N*_1/2_ = 12 × 10^3^ pulses (CU25). Photobleaching of red SAFD is the slowest we observe and *N*_1/2_ can only be extrapolated to be larger than 10^5^ pulses. Broadly speaking, these trends may reflect the expected increase in size of the aromatic fluorescent chromophore when going from blue to red emission. We may hypothesize that a higher number of vibrational degrees of freedom, associated with the increased size, should favor the safe dissipation of excess energy toward the solvent without permanent damage to the fluorophore. Besides, the enhanced photoresistance observed when moving from blue- to red-emitting CDs can also be seen as a consequence of their shorter lifetimes. In fact, a form of anticorrelation between photoresistance and lifetime may be expected if PB is due to a photochemical reaction in the excited state, as a longer lifetime implies in turn a longer permanence in this state. To address this point, we plotted the *N*_1/2_ values (which measure the degree of photostability) as a function of lifetime τ (Figure S7a) and of nonradiative coefficient *k*_NR_ = (1 – QY)/τ (Figure S7b). Excluding the hydrophobic sample CD49, a relation of the type *y* = *A*/*x*^α^, where α ≈ 2.35 and *A* is a constant, can be observed between the efficiency of the PB process and the lifetime of the pristine (nonphotobleached) CD samples.

Additionally, a dependence of the type *y* = *Ax*^α^ with α ≈ 1.65 can be observed between *N*_1/2_ and the calculated nonradiative decay coefficient of the same hydrophilic CD samples. The results suggest that nonradiative decay pathways bring about a protective effect on the excited state, by allowing the fluorophores to return to the ground state before the onset of destructive excited-state reactions. The deviation of sample CD49 from the observed trend is not surprising if we consider that these dots have a very different surface functionalization from all of the other CDs, and they are dispersed in a different solvent (CHCl_3_ vs H_2_O for all of the other CDs). The solvent, in particular, can have a crucial role in the chemical process leading to the darkening of the fluorophores. For this reason, the only meaningful comparison is among samples dispersed in the same solvent. The PB dependence on the solvent could be an interesting topic of future research but is beyond the goal of this work.

A consequence of these results that should be emphasized is that the higher resistance to PB comes at the expense of an increased nonradiative rate (Figure S7), which will tend to decrease the QY. For example, we observe that CZAU is >3 times more resistant to PB than CU25, but the QY of the latter is 1.7 times higher (Table S1). Whether a stronger photoresistance accompanied by lower QY is desired or not in practice depends on the specific application the CDs are intended for.

A notable feature of our results is that CDs with quasi-identical steady-state optical properties can display largely different PB dynamics. This is very clear looking at the samples CU25 and CZAU. Indeed, the almost perfect coincidence of CU25 and CZAU optical bands (Figure S8) strongly suggests an identical structure of the fundamental emitting unit responsible for the green emission in both cases. Compared with the literature, a possible candidate is HTTP, as reported by Kasprzyk and co-authors.^66^ However, the FLIP dynamics of these two samples are very different (Figure 4b and Table S3): the CZAU shows 3-fold more resistance to PB than the CU25 (*N*_1/2_ = 40 × 10^3^ pulses vs 12 × 10^3^ pulses, respectively), thus indicating they cannot be considered as fully equivalent. Even if we assume that CD fluorescence can be somehow traced back to HTTP molecular-like chromophore, our results strongly suggest a protective effect, to different extents, of the carbonaceous core to which the chromophore is attached to. The polymeric structure of CZAU appears to have a stronger protective effect against PB than the CU25. Based on the different synthetic routes (see the Materials and Methods section), we propose that in CZAU, the pre-formed fluorescent molecules (i.e., the citrazinic acid derivatives) are likely embedded within the structure and more shielded from photochemical reactions with the external environment, likely because of the formation of molecular aggregates before the thermal carbonization. In contrast, the CU25 fluorophores are probably attached to the external surface of a denser carbon core, and thus more exposed to detrimental influences from the environment. Indeed, a protective effect of the carbon core against PB effects was already proposed in previous studies of the PB of specific blue-emitting fluorophores.^67,68^ Anyway, the present FLIP experiments provide an efficient way to discriminate apparently identical CDs with respect to their photostability, which is an important functional property and is essential for viability in practical applications.

Finally, one may be tempted to correlate the damage induced by PB to the wavelength chosen for the bleach beam. Indeed, the blue-emitting samples, those in which the fluorescence faded the most, are also those for which the bleaching laser beam is in the UV. In contrast, the experiments on green- and red-emitting samples, which we found to be more and more resistant to PB, were performed with visible laser beams at increasingly longer wavelengths. For this reason, we carried out a comparative FLIP experiment for SAFD using a UV bleach beam of 266 nm and a probe beam of 532 nm, as opposed to λ_bleach_= λ_probe_ = 532 nm used in Figure 4. We chose SAFD for this comparative experiment because these CDs have shown the largest PB resistance in the previously described experimental conditions and because they show an additional UV absorption that excites the same red fluorescence.

The kinetics obtained are shown in Figure S9. Comparing it with the curves obtained for SAFD with bleach beam of 532 nm and for CT (bleach beam 355 nm as previously described), it is evident that UV light (266 nm) provides indeed a stronger PB of SAFD than visible light (532 nm), but SAFD remains more resistant of CT even when both are UV-irradiated. Additionally, it is worth noting that the resistance to PB, which is more than 10-fold higher upon visible excitation than UV excitation, does not scale with the ratio between the absorption coefficients at the two wavelengths, which only increases by a factor of 2 from 532 to 266 nm. In conclusion, although the PB wavelength plays a role in the rapidity of fluorescence fading, the main factor involved in the different PB efficiency displayed by the various samples is inherent to the sample itself, either due to the sample’s intrinsic structure or to the nature of the fluorophore, as further discussed below.

**Figure 5:**
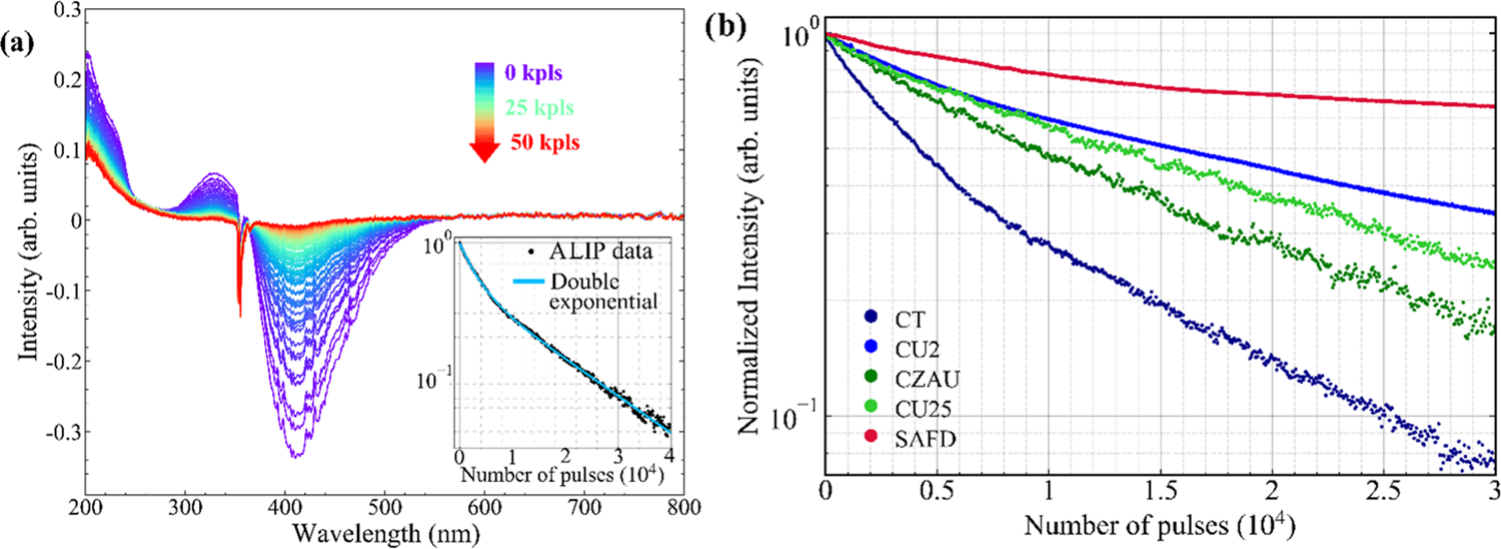
(a) Absorption loss in photobleaching (ALIP) spectra of CT sample. The color scale from blue to red indicates the increase of bleach pulses (355 nm) received by the sample. The negative signal consists of the fluorescence band induced by the bleach pulses at 355 nm and then interpreted by the spectrophotometer as an increase of transmitted light. The decay of absorbance intensity (integrated from 310 to 350 nm) is shown in the inset graph. The resulting double-exponential fit is also reported. (b) Comparison of ALIP decay curves of blue-, green-, and red-emitting CD samples.

After FLIP experiments, the CD photostability was deepened by investigating the absorption loss in photobleaching (ALIP), as described in the Materials and Methods section. Similarly to FLIP, CD samples were exposed to a prolonged series of high-intense bleach pulses at adequate wavelength, but in this case, the absorbance, rather than fluorescence, was monitored during the experiments. As an illustrative example of the ALIP experiment, the results obtained for the CT sample are shown in Figure 5a. The CT ALIP data show that the main absorption band peaked at 340 nm, responsible for the CT blue emission, consistently fades with increasing pulses number. This behavior suggests the photochemical breakdown of the initial blue-emitting fluorophore, which is converted into a nonfluorescent form. On the other hand, the shape of the residual unstructured absorption observed after several tens of thousands of pulses (red spectrum in Figure 5a) is consistent with the π–π* transition of the sp^2^ domains in the CDs core,^44^ after the emissive fluorophores have been entirely annihilated. However, such a residual UV absorption does not correspond to any fluorescence emission.

The dampening of the absorption band, as calculated from the integrated intensity in the range 330–350 nm, is reported in the inset of Figure 5a, and can be appropriately described by a double-exponential fit. A similar experiment was performed for all of the other samples. The results of the complete analysis performed on the ALIP curves (analogous to that shown for CT) are reported in Table S4, while a direct comparison of the ALIP decay curves is shown in Figure 5b.

As in the case of FLIP measurements, these data reveal significant heterogeneity among samples. Green-emitting CDs show a similar ALIP decay characterized by a single-exponential trend, and a half-life of about 10^4^ pulses, close to what we observed from FLIP. Also in this case, red CDs are the most resistant to PB, with a half-life of more than 3 × 10^4^ pulses. In contrast, the two blue CDs display very different behavior. CT shows a lower photostability and a shorter half-life of about 4000 pulses, in accordance with the result found in FLIP. In contrast, CU2 undergoes a relatively slow absorption PB despite its fluorescence PB was found to be very severe (please compare with Figure 4). As shown in Figure S10, no clear correlation between ALIP and FLIP data can be generally found. In particular, when plotting FLIP data vs ALIP data, the trends tend to fall below the line with a unitary slope, as evident in the case of the CU2 sample. This means that the fluorescence intensity decreases much faster than the absorption intensity, as can also be seen by directly comparing FLIP and ALIP curves (Figures 4b and 5b) for the same sample. For instance, the first 10^3^ bleaching pulses cause in CT a reduction of the fluorescence down to 50% of the initial value, while the absorption band at 360 nm only decreases to 20%. Similar results are found in the other samples. This result is not surprising and indicates that the PB converts the fluorophore into a nonfluorescent, but still absorbing form. Thus, the absorption cannot follow the same kinetics as the fluorescence because the nonfluorescent end product of the PB process still contributes to the absorption.

**Figure 6:**
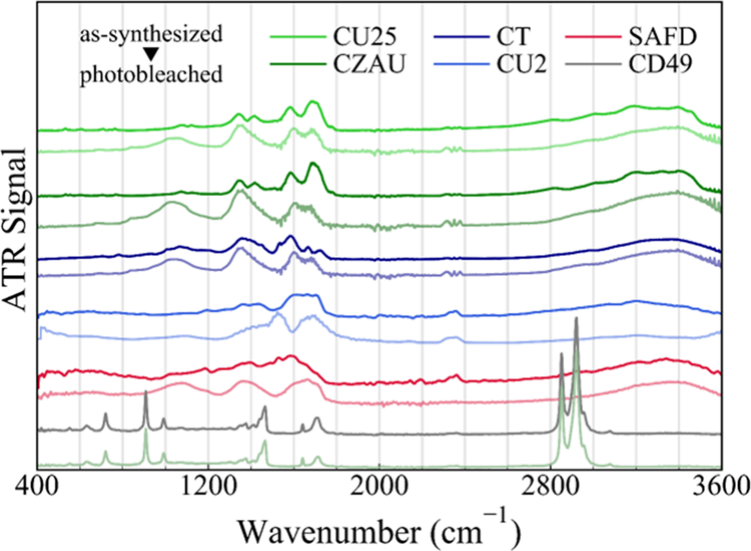
IR vibrational spectra of as-synthesized (top) and photobleached (bottom) samples performed by ATR. From top: CU25, CZAU, CT, CU2, SAFD, CD49.

Additional information about the structural modifications of CDs was obtained by the comparison between the ATR spectra of as-synthesized and photobleached samples (Figure 6). For the SAFD sample, to reach a significant degree of photodegradation, the PB has been carried out by administering 10^5^ pulses from a 10 mJ/pulse bleach beam (532 nm), whereas all of the other PB have been done with 10^5^ pulses of 1 mJ/pulse energy as in the rest of the paper. The FTIR spectra of photobleached samples generally show very marked changes. Most notably, the CZAU, CU25, CT, and SAFD spectra acquired after PB appear surprisingly similar to each other, irrespective of the specific sample and of the spectra of the pristine samples that were quite different from each other. After PB, they display three broad and unstructured features located at 1000–1100 cm^–1^ (C–O stretching, which may suggest photoinduced oxidation of the surface of the dot), 1300–1450 cm^–1^ (deformation of C–H, C–O–H, −CH_2_– and −CH_3_ groups), and 1600–1700 cm^–1^ (carboxylic and amide groups). At the same time, we observe the disappearance of some specific sharp features in the initial spectra, such as the NH bending (1415 cm^–1^) contributions, and the relatively sharp C=O contributions initially observed in some samples (1650–1700 cm^–1^). Similarly, in the region 3000–3500 cm^–1^, we observe the disappearance of sharp signals from C–H and N–H stretching initially observed in some samples.

Only samples CU2 and CD49 fall out of these general trends. The CU2 photobleached sample spectrum is very different from those already described, displaying a peak at around 1520 cm^–1^ (likely arising from N–H, C=N, C=C stretching) and no meaningful resonance in the range 2800–3500 cm^–1^. Only slight changes in the weak signals attributed to the C=O and amidic vibrations can be detected before and after the PB on the CD49 sample, both when photobleached with λ_pump_ = 355 nm (Figure 6) and λ_pump_ = 450 nm (spectrum not shown). The other signals, ascribed to the aliphatic C–H, C–C, and N–H vibrations do not undergo significant variations in intensity, as mainly attributed to the presence of residual ODE and HDA.

The portion of the ATR spectra wiped out by the PB could be attributed, in principle, to the emissive fluorophore. While the present data do not allow an unambiguous assignment of its molecular structure, the systematic disappearance of relatively sharp peaks associated with various functional groups is qualitatively consistent with the nature of the emissive fluorophores as small molecular-like units. For example, the contributions disappearing at 1665 and 1722 cm^–1^ in CT can be attributed to an amide and to an aldehyde functionality in the molecular structure of the blue emitter within CT carbon dots. On the other hand, excluding CD49, where we almost see no changes in the IR absorption upon PB, in all of the other cases, it is legitimate to attribute the residual spectrum after PB to the portion of CD structure, which does not participate in the fluorescence process. This contribution is weakly dependent on the type of CD, and it is likely ascribable to the carbon core, together with a variety of small, nonfluorescent surface functional groups. Interestingly, the unspecific nature of the final infrared absorption spectra, which are surprisingly similar in CU25, CZAU, CT, and SAFD, also supports the idea that PB proceeds through the chemical breakup of the initial fluorophore. For the CD49 sample, the peaks observed in the ATR spectra, being attributed either to hydrophobic aliphatic chains at the CD surface or to carboxylic/amide groups deriving from CD precursors, are not part of the molecular structure of emitting fluorophores. Therefore, fluorescence loss in the photobleached sample does not produce modification of the nonfluorescent group features observed in the ATR spectrum.

### Recovery after Photobleaching

**Figure 7:**
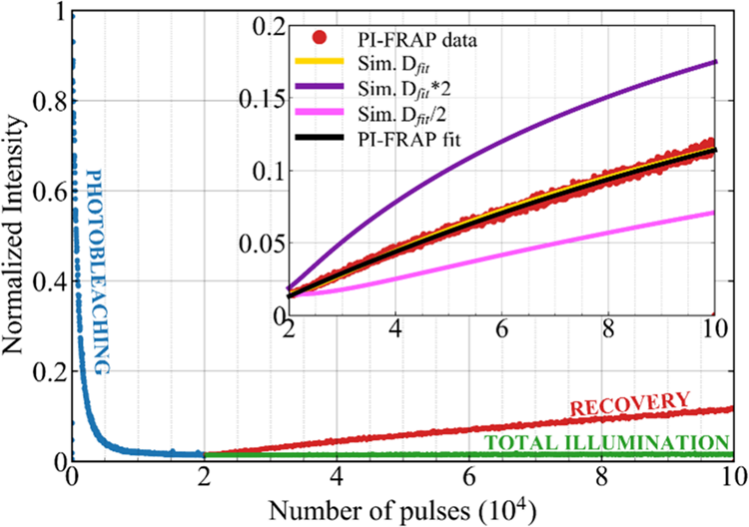
Fluorescence recovery after photobleaching (FRAP) of CT sample performed in total illumination (TI) and partial illumination (PI). The exponential fit of data and curves obtained from simulated diffusion are reported in the inset plot (representing a zoom of the main plot in the recovery region) and compared with simulations carried out using twice (*D*_*f*it_ × 2) or half (*D*_fit_/2) of the best fit diffusion constant.

Further information about the nature of CD fluorophores and their PB was obtained from the investigation of fluorescence recovery after photobleaching (FRAP) experiments, described in detail in the Materials and Methods section (Figure 1e). In Figure 7, we report the investigation of FRAP for CT as a representative case of all samples.

In the first stage of the experiment, the sample is subjected to a sequence of bleach pulses, leading to the PB of a large part of fluorescence (blue points). The experiment is either performed by illuminating the whole volume of the sample (total illumination, TI, Figure 1d) or only 25% of the total volume (partial illumination, PI, Figure 1e), while the remaining 75% is not photobleached. Then, the bleach laser is turned off, and we keep following the evolution of fluorescence intensity. In this second phase, a different behavior is found accordingly to the illumination geometry: in TI geometry, no fluorescence recovery is found in the observation stage (green points), whereas a progressive fluorescence recovery is revealed in PI geometry (red points). The lack of recovery in the first case indicates that the PB of our emitters, independently of their nature as free molecular fluorophores or fluorophores attached to CD cores, is irreversible and no intrinsic process (such as the inverse chemical reaction) is effectively able to restore their emission. Hence, the fluorescence recovery revealed in PI geometry can be uniquely attributed to the diffusion of nonphotobleached fluorophores coming back from the nonilluminated volume of the sample. Notably, the observed data trend clearly suggests that the recovery is not complete, and only affects a portion of the total population. Similarly to FLIP experiments, also in FRAP, we see no significant changes in the emission band shape during recovery.

FRAP experiments are often used in spectroscopy and microscopy to identify mobile fluorophores and measure their diffusion coefficient.^44,69^ The fluorescence recovery measured in FRAP is usually described by an exponential function *I*(*t*) = *M*_f_·(1 – exp(−*t*/τ))·*V*_corr_, where τ is the time scale of recovery, *M*_f_·*V*_corr_ is the saturation level of the recovery, and *M*_f_ estimates the mobile fraction of fluorophores. In our conditions, considering that the photobleached volume is one-quarter of the total volume, the recovery cannot be complete. This is taken into account by the parameter V_corr_ = (1–V_PB_/ V) = 0.75, where V is the total volume of sample and V_PB_ is the illuminated volume that undergoes the PB. From the best fit curve, we obtain a mobile fraction *M*_f_ ≈ 37 ± 2%, and a time scale τ ≈ 4.1 h. The first parameter indicates that the fluorophores of CT are constituted by two kinds of species having the same spectroscopic characteristics but different diffusivity since 1 – *M*_f_ ≈ 63% of fluorophores do not participate at all in the diffusion revealed by FRAP, at least in the time scale explored by our experiments. Besides, based on the random walk description of molecular Brownian motion, we can relate the time scale of fluorescence recovery to the mass diffusivity through the equation *D* = *x*^2^/2τ. Using the height of the liquid contained in the cuvette (∼0.5 cm, see Figure 1e) as a value for *x*, we obtain the value *D*_fit_ ≈ 8.5 × 10^–10^ m^2^/s. From the Stokes–Einstein equation *D* = *kT*/6πη*r* (where η is the viscosity of water), we then estimate the hydrodynamic radius of diffusive objects equal to *r* = 0.27 ± 0.01 nm. This value is 1 order of magnitude smaller than the mean size of carbon nanoparticles measured by TEM (2.0 nm or more) but compatible with a typical hydrodynamic radius of a small molecular fluorophore in solution. Thus, the small molecular fluorophores are responsible for 37% of the observed fluorescence. In contrast, carbon nanoparticles, which account for the remaining 63%, are expected to undergo diffusion on much longer time scales than those accessible by our experiment; hence, they do not contribute to the diffusional recovery we observe in Figure 7. Considering the size of our CDs, typically in the range of several nanometers, their D coefficient should be of the order of 10^–11^ m^2^/s, both in water and in CHCl_3_; hence, their diffusion should typically be 1 order of magnitude slower than molecular fluorophores.

The experiment was repeated for all of the other CDs, and the results are shown in Table S5. In CU2, the other blue-emitting CDs, we found *r* = 0.23 ± 0.03 nm and a mobile fraction of ≈37%. For green-emitting CDs, we found a comparable size of the mobile units (≈0.2 nm), but substantially lower mobile fractions between each other: ≈38% for CZAU and ≈20% for CU25, respectively (see Table S5). No recovery at all was observed for red CDs, suggesting that the size of the emitting units is significantly larger than the blue or green mobile fluorophores. Besides, the very limited amount of PB induced by irradiation on SAFD makes it very difficult to carry out a FRAP experiment similar to Figure 7, which is founded on the possibility of achieving a strong PB in the first 2 × 10^3^ s (or 2 × 10^4^ pulses).

We further checked the interpretation of FRAP as a consequence of diffusion by numerically solving the mass transport equation ∂Φ/∂*t* = −∇·*J*, where Φ is the concentration of the diffusing species and *J* is the current flow density, in the geometry used in our experiment. Our simulation predicts the time-dependent number of fluorescent objects within the probed volume, using as input parameters the diffusion coefficient only, and assuming an initial fluorescence of zero in the probed volume just after PB. As shown in the inset plot of Figure 7, the simulated curve (yellow line in the inset) features an excellent agreement with FRAP data when using the value *D*_fit_ = 0.85 × 10^–9^ m^2^/s obtained by the simple exponential fit described above. These results further support the interpretation of a FRAP analysis based on the diffusion process. Finally, the accuracy of such a procedure was evaluated by comparing the curves obtained from two further simulations in which we varied the diffusion coefficient to twice and half the best value.

It is also instructive to compare the results obtained for the CD samples with what was observed in a pure molecular dye, i.e., CZAc, whose optical properties (Figure S5), as already mentioned, are very similar to those of the CU2 and CT samples. Despite the fluorescence emitted by CZAc and CU2 are virtually indistinguishable, the FLIP and the FRAP measurements reveal a strong difference between the two samples: in fact, the FLIP photobleaching rate of the CU2 sample is markedly slower than that of the dye (Figure S11a and Tables S2 and S3, *N*_1/2_ = 1.3 and 0.3, respectively, for CU2 and CZAc), which seems to confirm the protective effect of the carbonaceous core as argued above.

Besides, the recovery in the dye sample is significantly faster (Figure S11b). In fact, the diffusion of CZAc is so rapid compared to the time scale of the experiment that the fluorescence loss during the first stage of the FRAP (during which the sample is subjected to the bleach pulses) appears deceptively less pronounced (see the comparison between CU2 and CZAc curve in Figure S11). The results of the analysis, reported in Table S2, provided for CZAc a value of *M*_f_ = 100%, which indicates the complete homogenization of the spatial distribution of nonphotobleached molecules after τ ≈ 2.7 h from the end of irradiation. These results confirm that in the dye sample, the fluorescence units are all small molecules, as expected. By contrast, they also imply that the majority (i.e., 1 – *M*_f_) of the emitting fluorophores in the CU2 sample are incapable of diffusion on these time scales. Similar conclusions can be drawn for all CD samples since *M*_f_ ranges from 17 to 38%, as shown in Table S5.

Thus, our results allow gaining a deeper insight into the fluorescence of CDs, confirming several of our previous hypotheses. By considering the full spectroscopic similarity between the nonphotobleached samples and the mobile fraction revealed in FRAP experiment, we deduce that the fluorophore responsible for emission in each CD sample can be always attributed to a single molecular species, specifically determined by the synthesis. However, for a given type of CD, the fluorophores are constituted by two distinct groups featuring different mobility. The high-mobility fraction revealed by FRAP can be attributed to free molecular fluorophores in solution, based on the characteristic size we found. Conversely, the low-mobility fraction corresponds to larger and heavier objects, which do not diffuse in the time scale of our FRAP experiments. The latter can clearly be attributed to the same fundamental fluorophores, stably linked to, or embedded into CDs.

Interestingly, the relative proportions between these two species (see Table S5) are highly dependent on the type of CDs. On the other hand, it is worth noting that our findings may be also influenced by the degree of purity obtained by the different synthesis procedures, as the relative proportion of the mobile fraction is certainly influenced by the effectiveness of the purification step in removing it from the sample.

The different weights of the mobile fractions are in line with the results obtained by FLIP and ALIP experiments, where the inverse trend was found for the resistance of the PB. In fact, our data imply a protective effect of CD cores on the PB of the emitting fluorophore, which should tend to increase whenever most of these units are effectively embedded into the structure of CDs. Indeed, the protective effect that we propose is directly highlighted by comparing CZAc and CU2 samples (Figure S11) and more generally confirmed by the observation that even CDs displaying almost identical emission properties are observed to respond very differently to PB.

Methodologically, our investigation confirms that the fluorescence of typical bottom-up CD samples is indeed affected by the presence of small molecular units, as claimed by several studies.^22,32−38^ However, it also provides clear evidence that these molecular units are, for a large portion, incorporated into the structure of CDs or stably adsorbed on their surfaces. Discriminating free fluorophores from embedded ones is not only a matter of classification, if one considers the functional effects potentially induced by the interactions between the emissive fluorophore and the carbon cores. Indeed, the interactions between molecular fluorophores and CDs bear relevant consequences on both their photostability and their fundamental optical properties (such as observed through the nonexponential decay and variations of QY from free to embedded form). More generally, the combination of FLIP, ALIP, and FRAP studies provides a route to disentangle free molecules from fluorophores adsorbed/embedded/bound on CDs in a way that would be impossible based on optical data alone.

## Conclusions

We carried out a systematic study of the PB of CD fluorescence using different techniques capable to follow in situ the evolution of their optical spectra during high-intensity irradiation. Comparative experiments on a palette of different CDs emitting across the whole visible spectrum reveal various trends in their response to PB and important details on their emission mechanisms and fundamental nature. A certain degree of PB is observed on all samples, mostly likely progressing through the photoinduced breakup of the emissive molecular units. However, the resistance to high-intensity irradiation is highly sample-dependent, and even CDs that display almost identical absorption and emission properties can respond very differently to PB. As a general trend, the resistance to PB tends to increase from blue to red emitters and inversely correlates with the excited-state lifetime. Overall, our results strongly suggest that the interactions between fluorescent units and carbonaceous CD cores not only affect the excited-state decay properties of the former but also have a protective effect against PB. In the case of blue-emitting CDs, this view is directly confirmed by comparing PB between CDs and citrazinic acid molecules. For all types of CDs, the kinetics of PB display a multiplicity of rates, associated with a variety of possible configurations of the emissive fluorophore on the surface of CDs, or embedded into it. Finally, PB experiments conducted in a nonuniform irradiation geometry allow us to study the diffusion properties of the fluorescent units. Based on these data, we demonstrate a method to quantify the portion of CD emission coming from free molecular units from fluorescence associated with fluorophores that are stably embedded into the structure of the dots. In this way, we show that embedded fluorophores are always a majority, in all types of CDs we studied.

Overall, our approach demonstrates a route to systematically study the factors controlling the resistance to PB of CDs with different structures. Besides, our results reveal several unknown details on the PB processes of CDs and provide important information on the fundamental properties of their emissive units.

## Supporting Information Available

TEM images of all of the CDs, absorption spectra, further optical characterizations including citrazinic acid, time-resolved fluorescence decay data, lists of parameters obtained from the different fits, FLIP graphics, photoresistance as a function of lifetime and nonradiative decay coefficient, FLIP curves compared at different bleaching wavelength, FLIP-ALIP correlation, and FLIP-FRAP comparisons (PDF)
