# Supplementary material for: Photobleaching and Recovery Kinetics of a Palette of Carbon Nanodots Probed by In Situ Optical Spectroscopy
Source: ACS Appl Mater Interfaces. 2022 Jul 27;14(31):36038–51. doi: 10.1021/acsami.2c09496 (PMC9376924; doi:10.1021/acsami.2c09496)
Supplement: Supplementary file 1 — am2c09496_si_001.pdf [file am2c09496_si_001.pdf]

Supporting Information

Photobleaching and recovery kinetics of a palette of carbon nanodots probed by in situ optical spectroscopy.

Angela Terracina[1],, Angelo Armano[1], Manuela Meloni[2], Annamaria Panniello[3], Gianluca Minervini[3,4], Antonino Madonia[3], Marco Cannas[1], Marinella Striccoli[3]†, Luca Malfatti[2]††, Fabrizio Messina[1].*

(1) Dipartimento di Fisica e Chimica, Università degli Studi di Palermo, Via Archirafi 36, 90123 Palermo, Italy;

(2) Department of Chemistry and Pharmacy, Laboratory of Materials Science and Nanotechnology, CR-INSTM, University of Sassari, Via Vienna 2, 07100, Sassari, Italy;

(3) CNR-IPCF-Bari Division, c/o Chemistry Department, and Chemistry Department, University of Bari "Aldo Moro", Via E. Orabona 4, 70126 Bari, Italy;

(4) Department of Electrical and Information Engineering, Polytechnic of Bari, Via E. Orabona, 4, 70126 Bari, Italy.

† m.striccoli@ba.ipcf.cnr.it; ††luca.malfatti@uniss.it; *fabrizio.messina@unipa.it

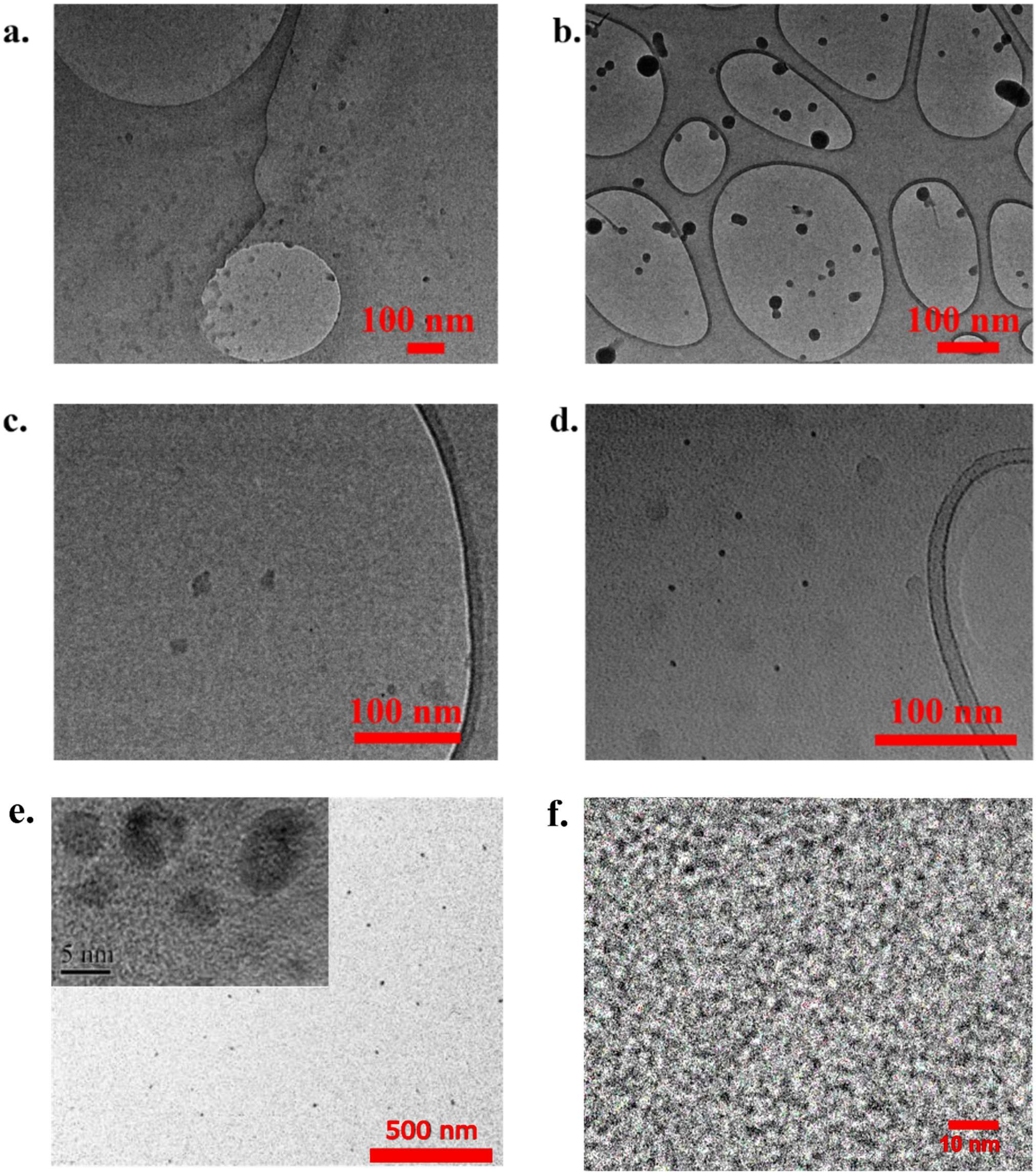

Figure S1 *- Representative TEM images of CU2 (a), CU25 (b), CZAU (c), SAFD (d), CT (e) and CD49 (f).*

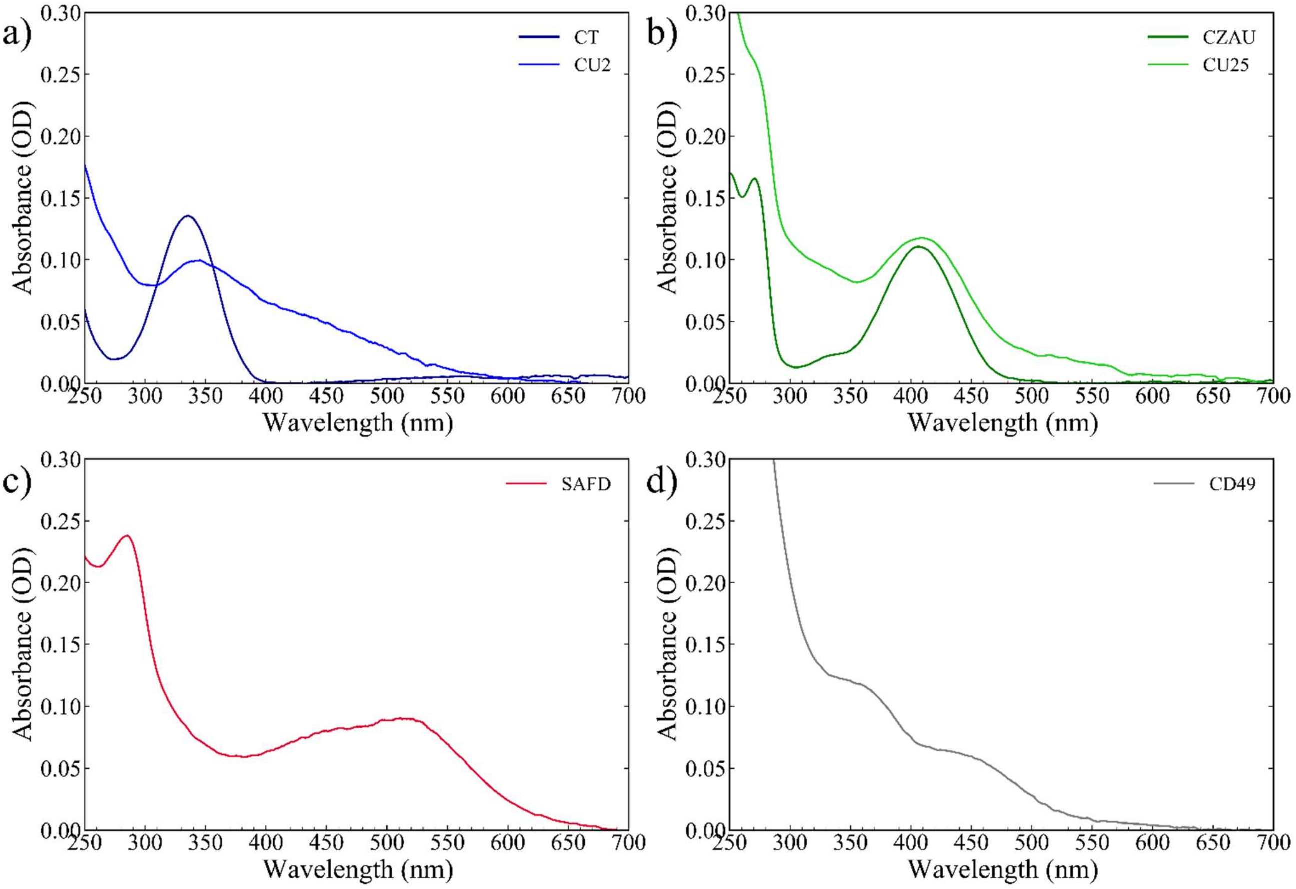

Figure S2: *(a)-(d) Absorption spectra of all the CD samples investigated in this study.*

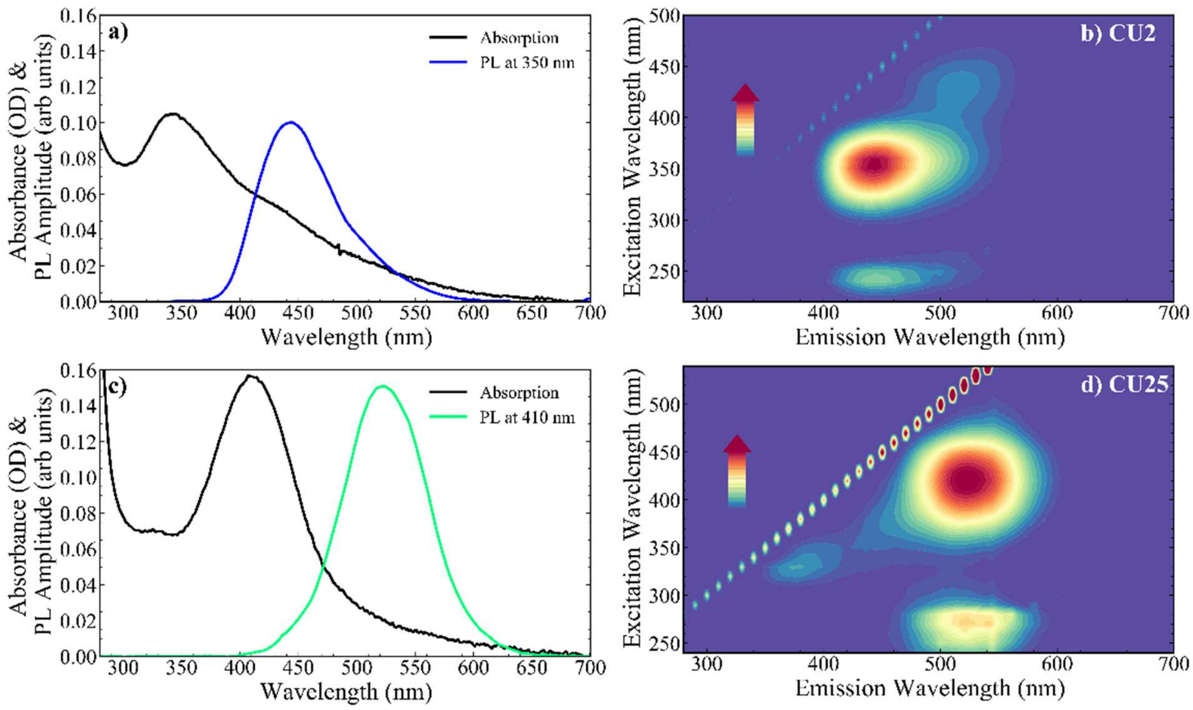

Figure S3 *- Optical characterization of additional samples: CU2 and CU25. Absorption and fluorescence (excited at absorption peak) spectra of* ***(a)*** *CU2 and* ***(c)*** *CU25; 2D excitation-emission fluorescence intensity map of* ***(b)*** *CU2 and* ***(d)*** *CU25.*

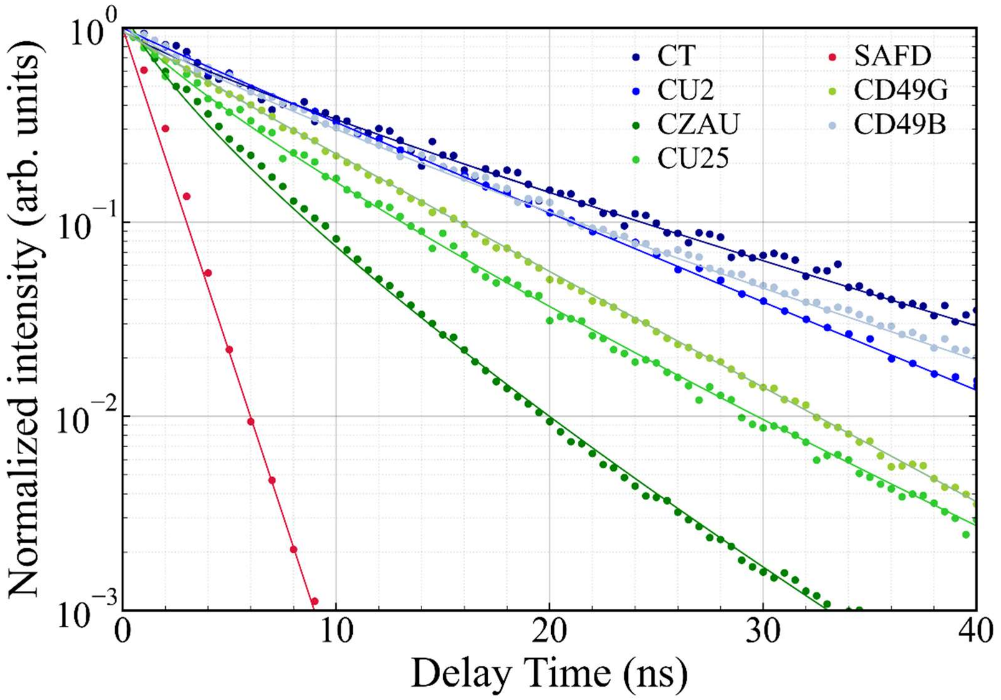

Figure S4 – *Time-resolved fluorescence decay data collected from all the CD samples and corresponding best-fitting curves.*

Table S1 – *Optical characterization of the investigated CD samples: excitation and emission peak wavelength, lifetimes and β stretching factors, as obtained by least-square fitting of time-resolved photoluminescence data with a stretched exponential function, quantum yield estimates.*

Sample	λ_{exc} (nm)	λ_{em} (nm)	τ (ns)	β	QY
**CT**	340	420	9.5(5)	0.87(5)	57(6) %
**CU2**	350	430	8.9(4)	0.97(5)	32(3) %
**CZAU**	410	530	2.6(1)	0.77(5)	10(1) %
**CU25**	410	530	4.5(2)	0.82(5)	17(2) %
**SAFD**	530	580	1.3(1)	1.00(5)	4(1) %
**CD49B**	370	430	7.7(4)	0.84(5)	46(5) %
**CD49G**	450	560	6.8(3)	0.97(5)	7(1) %

Table S2 – *Results obtained for CZAc in the different experiments carried out in the manuscript.*

**General characterization**	**λ_{exc} (nm)**	**λ_{em} (nm)**	**Lifetime τ (ns)**	**β (Stretching)**	**QY (%)**	
	355	440	6.0(3)	1.00(5)	19(2)	
**FLIP Results**	**λ_{exc} (nm)**	**$N_{1/2}$ ($\cdot 10^3$)**	**Exponentials**	**N_1($\cdot 10^3$)**	**N_2($\cdot 10^3$)**	**N3($\cdot 10^3$)**
	355	0.3(1)	3	0.9	6.4	>10^5
**FRAP Results**	**λ_{phb} (nm)**	**M_f (%)**	**τ (h)**	**D ($\cdot 10^{-10}$ m^2/s)**	**r (nm)**	
	355	100 (10)	2.7	13.1	0.18(6)	

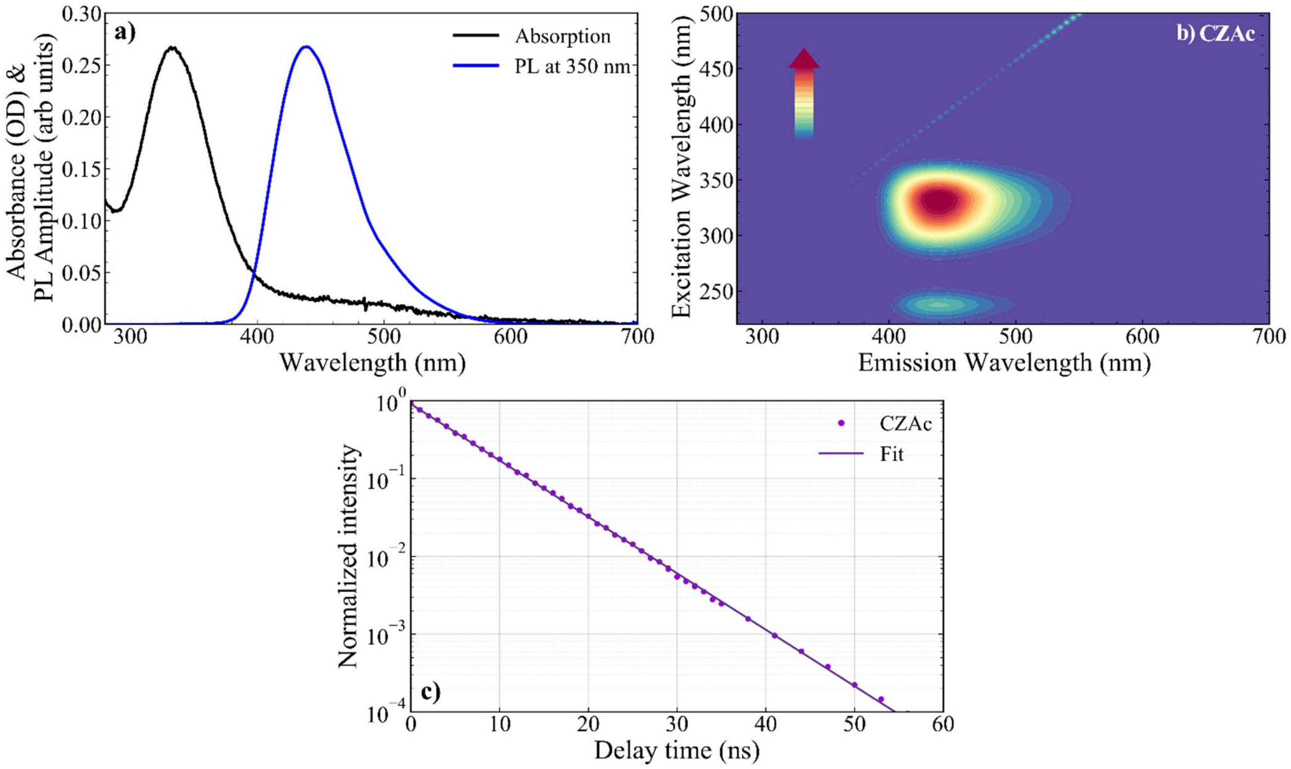

Figure S5 – *Optical characterization of CZAc: (a) absorption and fluorescence (excited at absorption peak) spectra; (b) 2D excitation-emission fluorescence intensity map; (c) fluorescence decay in time-resolved photoluminescence.*

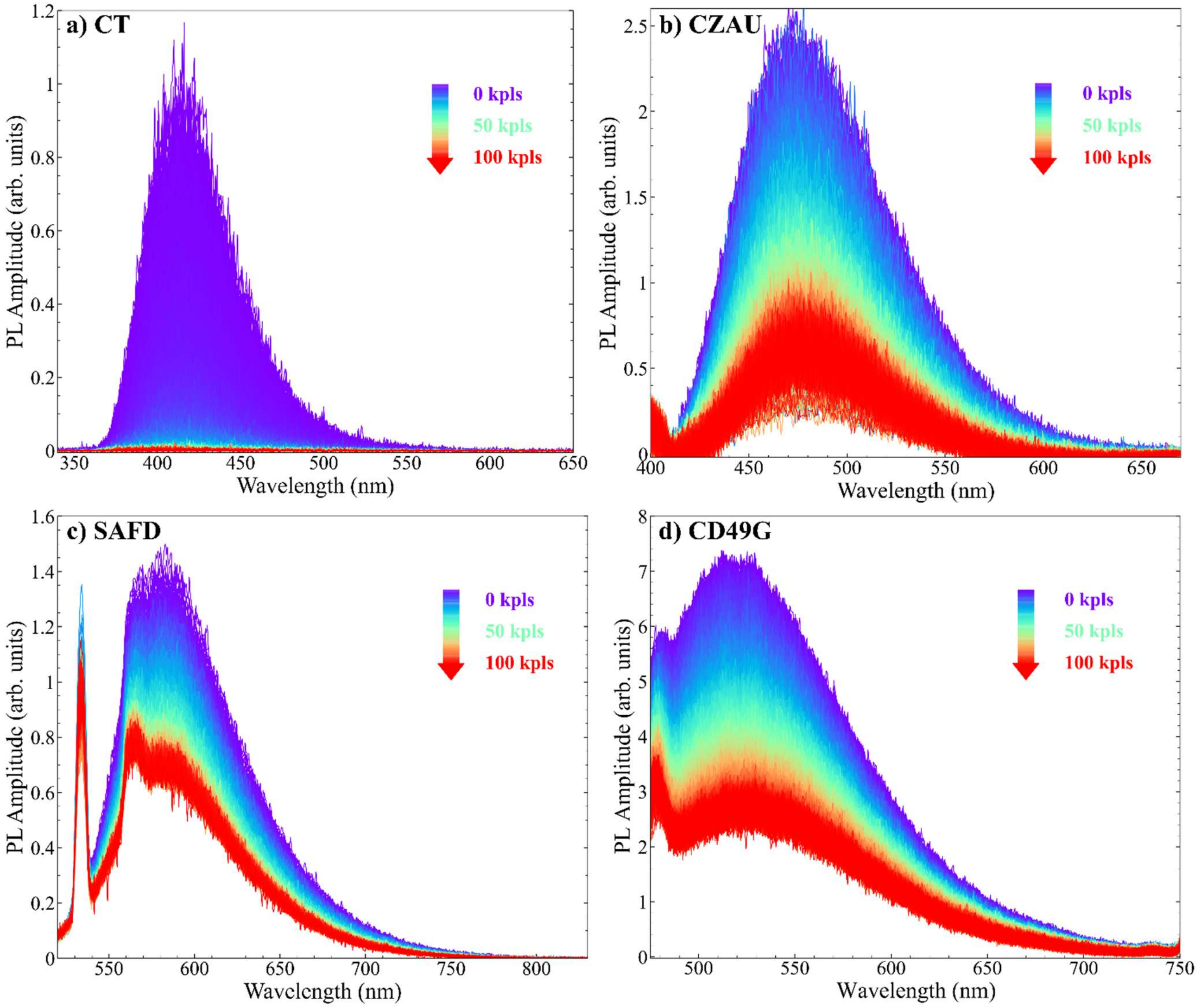

Figure S6 *- Fluorescence loss in photobleaching (FLIP) experiment on representative sample: (a) CT, (b) CZAU, (c) SAFD and (d) the green-yellow band of CD49. The spectra are shown from virtually 0 pulses (violet) to* 10^5 *pulses (red).*

Table S3 *– Results obtained from the analysis of the FLIP experiments: the parameter $N_{1/2}$ expressing the number of bleaching pulses needed to halve the initial fluorescence intensity, the number of exponential terms necessary to properly fit the experimental data and the three values of N_i characterizing the multiexponential fit*

Sample	λ_{exc} (nm)	$N_{1/2}$ (10^3)	#exp	N_1($\cdot 10^3$)	N_2($\cdot 10^3$)	N_3($\cdot 10^3$)
**CT**	355	1.2(1)	3	1.8 (weight=80.8%)	7.2 (19%)	106 (0.2%)
**CU2**	355	1.3(2)	3	2.1 (11%)	8.9(87%)	81(2)
**CZAU**	410	40(4)	1	45		
**CU25**	410	12(2)	3	1 (18%)	21 (64%)	123 (18)
**SAFD**	532	>100	1	33		
**CD49B**	355	0.4(2)	3	1.4 (9%)	7 (89%)	62 (2%)
**CD49G**	450	80(5)	2	16 (17%)	160 (83%)	

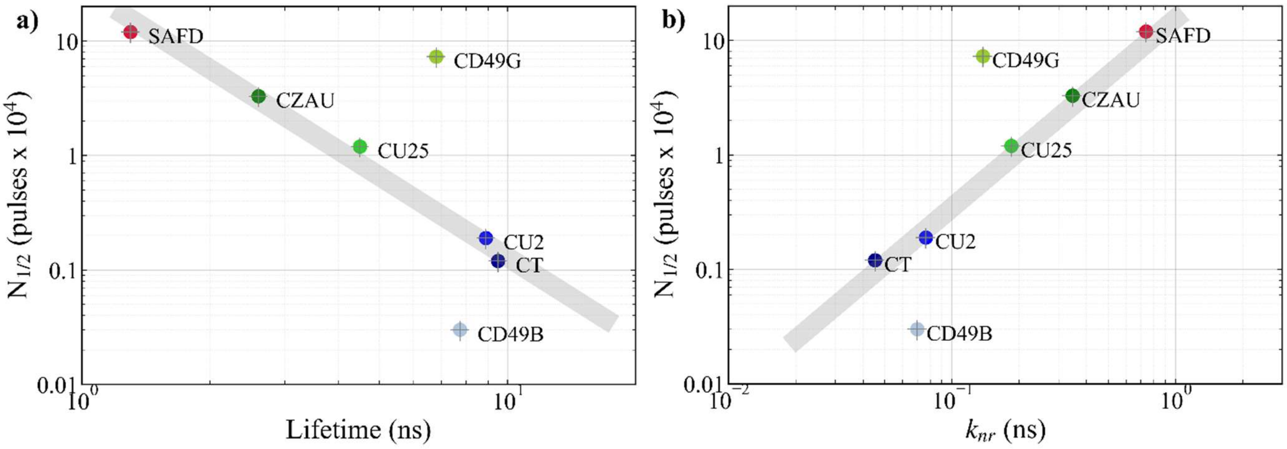

Figure S7 *– Plot displaying the correlation between $N_{1/2}$ values as a function of lifetime τ (a) and the non-radiative decay coefficient k_{NR} (b). The grey lines in both panels are a guide to the eye to highlight the relation between the two quantities. In panel (a) the equation is of the type $y=m_1 \cdot 1/x^k$ with k=2.35 whereas in panel (b) the equation is $y=m_2 \cdot x^k$ with k=1.65.*

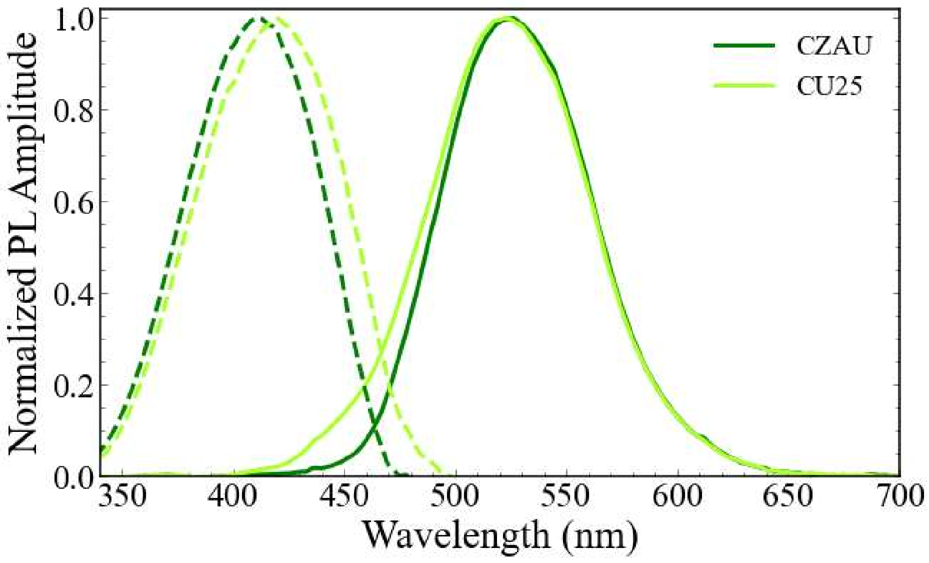

Figure S8 *- Fluorescence spectra of CZAU (dark green) and CU25 (light green) excited at 410 nm (straight lines) and excitation spectra with emission at 525 nm (dashed lines). The spectra are normalized for the peak intensities.*

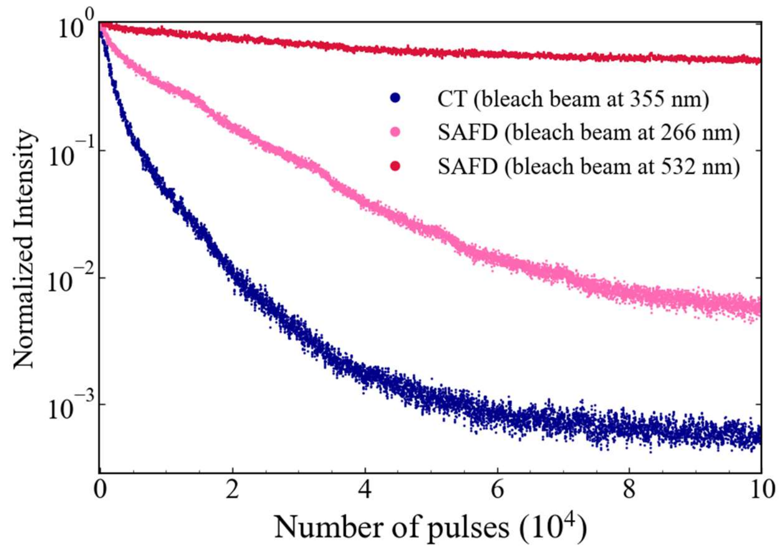

Figure S9 *- Comparison between FLIP curves for samples CT bleached by a 355 nm beam (blue curve), SAFD bleached by a 266 nm beam (pink curve) and SAFD bleached by a 532 nm beam (red curve).*

Table S4 *- Results obtained from the analysis of the ALIP experiments: the parameter $N_{1/2}$ expressing the number of pulses needed to halve the initial fluorescence intensity, the number of exponential terms necessary to properly fit the experimental data and the values of N_i characterizing the multiexponential fit*

**Sample**	**λ_{ecc} (nm)**	**$N_{1/2}$ ($\cdot 10^3$)**	**exp**	**N_1($\cdot 10^3$)**	**N_2($\cdot 10^3$)**
**CT**	340	4.1(1)	2	2.8 (weight=58%)	15 (42%)
**CU2**	350	15(1)	2	5 (36%)	45 (64%)
**CZAU**	410	10(1)	1	2.9 (19%)	19 (81)
**CU25**	410	13(1)	1	0.7 (7%)	22 (93%)
**SAFD**	530	37(5)	2	9.4 (34%)	261 (66%)

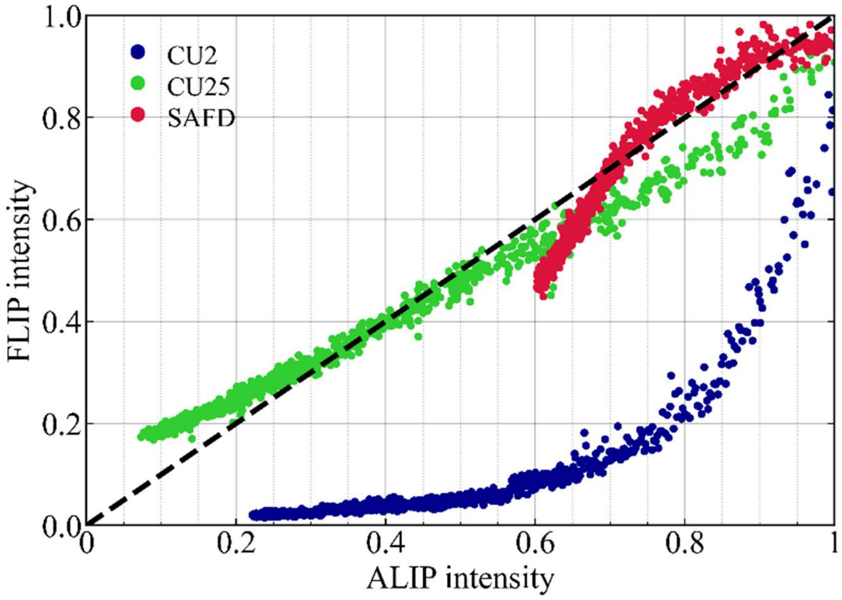

Figure S10*- FLIP-ALIP correlation plot for selected samples.*

Table S5 *- Results obtained from the analysis of the FRAP experiments: M_f is the saturation level of the recovery, thus representing the mobile fraction of fluorophores, τ is the time scale of the recovery, D is the mass diffusivity and r is the estimated radius of the diffusive objects.*

Sample	λ_{probe} (nm)	M_f (%)	τ (h)	D ($\cdot 10^{-9}$ m^2/s)	r (nm)
**CT**	340	37(2)	4.1	0.85	0.27(1)
**CU2**	350	37 (7)	3.4	1.01	0.23(3)
**CZAU**	410	38 (9)	2.8	1.26	0.18(6)
**CU25**	410	19 (7)	2.6	1.34	0.18(6)
**CD49B**	370	17 (7)	0.7	5.0	0.08(4)
**CD49G**	450	33 (6)	1.8	1.95	0.21(6)

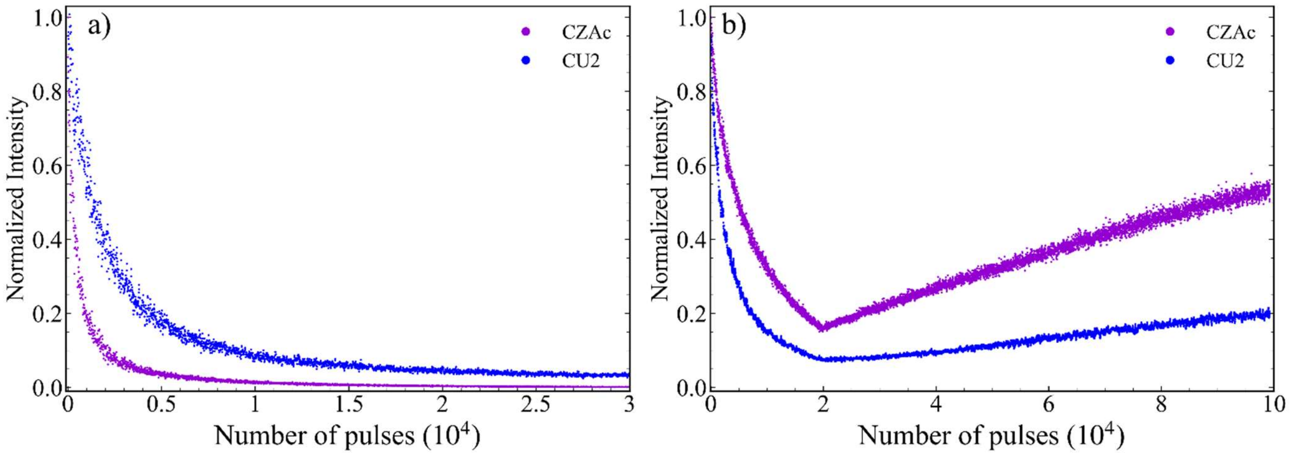

Figure S11 *– Comparison between (a) FLIP curves and (b) FRAP curves for the CD sample CU2 (blue curves) and the dye CZAc (violet curves).*
